# Acupuncture for Chronic Radiation-Induced Xerostomia in Head and Neck Cancer: A Multicenter Randomized Clinical Trial

**DOI:** 10.1001/jamanetworkopen.2024.10421

**Published:** 2024-05-13

**Authors:** Lorenzo Cohen, Suzanne C. Danhauer, M. Kay Garcia, Emily V. Dressler, David I. Rosenthal, Mark S. Chambers, Andrew Cusimano, W. Mark Brown, Jewel M. Ochoa, Peiying Yang, Joseph S. Chiang, Ora Gordon, Rhonda Crutcher, Jung K. Kim, Michael P. Russin, Joshua Lukenbill, Mercedes Porosnicu, Kathleen J. Yost, Kathryn E. Weaver, Glenn J. Lesser

**Affiliations:** 1 Department of Palliative, Rehabilitation, and Integrative Medicine, The University of Texas MD Anderson Cancer Center, Houston; 2 Department of Social Sciences & Health Policy, Wake Forest University School of Medicine, Winston-Salem, North Carolina; 3 Department of Biostatistics & Data Science, Wake Forest University School of Medicine, Winston-Salem, North Carolina; 4 Department of Radiation Oncology, The University of Texas MD Anderson Cancer Center, Houston; 5 Department of Dental Oncology, The University of Texas MD Anderson Cancer Center, Houston; 6 Department of Anesthesiology, The University of Texas MD Anderson Cancer Center, Houston; 7 Disney Family Cancer Center, Department of Integrative Medicine, Providence St Joseph Medical Center, Burbank, California; 8 Medical Oncology and Hematology, Kaiser Permanente Diablo Service Area, Martinez, California; 9 Iowa-Wide Oncology Research Coalition NCORP, Des Moines; 10 Section on Hematology & Oncology, Department of Internal Medicine, Wake Forest University School of Medicine, Winston-Salem, North Carolina; 11 Cancer Research Consortium of West Michigan NCORP, Spectrum Health at Butterworth Campus, Grand Rapids

## Abstract

**Importance:**

Patients with head and neck cancer who undergo radiotherapy can develop chronic radiation-induced xerostomia. Prior acupuncture studies were single center and rated as having high risk of bias, making it difficult to know the benefits of acupuncture for treating radiation-induced xerostomia.

**Objective:**

To compare true acupuncture (TA), sham acupuncture (SA), and standard oral hygiene (SOH) for treating radiation-induced xerostomia.

**Design, Setting, and Participants:**

A randomized, blinded, 3-arm, placebo-controlled trial was conducted between July 29, 2013, and June 9, 2021. Data analysis was performed from March 9, 2022, through May 17, 2023. Patients reporting grade 2 or 3 radiation-induced xerostomia 12 months or more postradiotherapy for head and neck cancer were recruited from community-based cancer centers across the US that were part of the Wake Forest National Cancer Institute Community Oncology Research Program Research Base. Participants had received bilateral radiotherapy with no history of xerostomia.

**Interventions:**

Participants received SOH and were randomized to TA, SA, or SOH only. Participants in the TA and SA cohorts were treated 2 times per week for 4 weeks. Those experiencing a minor response received another 4 weeks of treatment.

**Main Outcomes and Measures:**

Patient-reported outcomes for xerostomia (Xerostomia Questionnaire, primary outcome) and quality of life (Functional Assessment of Cancer Therapy–General) were collected at baseline, 4 (primary time point), 8, 12, and 26 weeks. All analyses were intention to treat.

**Results:**

A total of 258 patients (201 men [77.9%]; mean [SD] age, 65.0 [9.16] years), participated from 33 sites across 13 states. Overall, 86 patients were assigned to each study arm. Mean (SD) years from diagnosis was 4.21 (3.74) years, 67.1% (n = 173) had stage IV disease. At week 4, Xerostomia Questionnaire scores revealed significant between-group differences, with lower Xerostomia Questionnaire scores with TA vs SOH (TA: 50.6; SOH: 57.3; difference, −6.67; 95% CI, −11.08 to −2.27; *P* = .003), and differences between TA and SA (TA: 50.6; SA: 55.0; difference, −4.41; 95% CI, −8.62 to −0.19; *P* = .04) yet did not reach statistical significance after adjustment for multiple comparisons. There was no significant difference between SA and SOH. Group differences in Functional Assessment of Cancer Therapy–General scores revealed statistically significant group differences at week 4, with higher scores with TA vs SOH (TA: 101.6; SOH: 97.7; difference, 3.91; 95% CI, 1.43-6.38; *P* = .002) and at week 12, with higher scores with TA vs SA (TA: 102.1; SA: 98.4; difference, 3.64; 95% CI, 1.10-6.18; *P* = .005) and TA vs SOH (TA: 102.1; SOH: 97.4; difference, 4.61; 95% CI, 1.99-7.23; *P* = .001).

**Conclusions and Relevance:**

The findings of this trial suggest that TA was more effective in treating chronic radiation-induced xerostomia 1 or more years after the end of radiotherapy than SA or SOH.

**Trial Registration:**

ClinicalTrials.gov Identifier: NCT02589938

## Key Points

**Question:**

Is acupuncture an effective treatment to reduce symptoms of radiation-induced xerostomia once it becomes chronic after the completion of radiotherapy for head and neck cancer?

**Findings:**

In this randomized clinical trial including 258 patients with head and neck cancer, true acupuncture was effective at improving symptoms of radiation-induced xerostomia and overall quality of life (QOL) compared with standard oral hygiene. Although there was some suggestion of a sham effect, the benefits of sham treatment were minimal and not associated with improvements in overall QOL.

**Meaning:**

The findings from this trial suggest that use of real acupuncture reduces chronic radiation-induced xerostomia and leads to improvement in QOL.

## Teaser

This randomized clinical trial evaluates the use of acupuncture in the treatment of xerostomia in patients with head and neck cancer who received radiotherapy.

## Introduction

By the end of radiotherapy, more than 50% of patients with head and neck cancer experience hyposalivation with the subjective sensation termed radiation-induced xerostomia (RIX)^1,2,3^; its influence on quality of life among patients with cancer is well established.^4,5^ Patients experience decreased or total lack of saliva secretion leading to pain and difficulty speaking, chewing, swallowing, and sleeping, as well as taste aberration, insufficient nutritional intake, weight loss, caries, loss/deterioration of dentition, and gingivitis.^6^ Despite some success with cytoprotection (eg, amifostine)^7^ and physical techniques designed to reduce salivary gland exposure during radiotherapy,^8^ acute and chronic RIX still occur^9^ with no reliable treatment.^10^

A number of randomized and observational studies have found acupuncture can stimulate saliva flow and improve patient-reported outcomes among participants with RIX.^11,12,13,14,15,16,17,18,19,20,21,22^ These studies were conducted by different investigators, in multiple countries, using different acupuncture points, yet all produced similar positive results. One study even demonstrated long-term effects (>3 years) on saliva production,^13^ and several studies^14,21,23,24^ found that acupuncture can prevent RIX when given concurrently with radiotherapy. However, prior trials lacked blinding or sham controls and/or had small sample size.

A large, 2-center (US and China), 3-arm, sham-controlled, phase 3 clinical trial of acupuncture to prevent RIX when provided concurrently with radiotherapy was conducted.^17^ This trial found that xerostomia was significantly lower in the true acupuncture (TA) group compared with standard oral hygiene (SOH) and marginally lower than a sham acupuncture (SA) group. To our knowledge, there have been no phase 3, multicenter trials to examine the effects of acupuncture to treat chronic RIX. The present randomized, phase 3, blinded, sham-controlled, multicenter trial examined whether TA could symptomatically improve RIX in patients with head and neck cancer with moderate or severe RIX.^25^

## Methods

### Study Design and Participants

The study was launched July 2013 under the oversight of the MD Anderson Community Clinical Oncology Program Research Base. Twenty-eight participants were recruited across multiple centers from July 29, 2013, to August 20, 2015. The trial was subsequently transferred to the Wake Forest National Cancer Institute Community Oncology Research Program (NCORP) Research Base where the trial continued from April 7, 2016, through June 9, 2021 (protocol available in Supplement 1). The trial was approved by the institutional review boards of the Wake Forest University School of Medicine, The University of Texas MD Anderson Cancer Center, and each NCORP site where participants were enrolled and provided informed consent; they did not receive financial compensation. The study follows the Consolidated Standards of Reporting Trials (CONSORT) reporting guideline for RCTs.

Eligibility criteria included (1) diagnosis of head and neck cancer; (2) aged 18 years or older; (3) ability to read, write, and understand English; (4) received only first-line bilateral external-beam radiotherapy with curative intent 12 or more months prior to enrollment; (5) grade 2 or 3 xerostomia per Radiation Therapy Oncology Group scale known to be due to radiotherapy; (6) had anatomically intact parotid glands and at least 1 submandibular gland; (7) had never received acupuncture for xerostomia; and (8) Eastern Cooperative Oncology Group performance status of 0 to 2. Exclusion criteria included (1) history of xerostomia, Sjögren syndrome, or other illness known to affect salivation prior to radiotherapy; (2) suspected or known closure of salivary gland ducts on either side; (3) currently receiving or planning to receive other xerostomia treatment; (4) receiving (past 30 days) or planning to receive any investigational drug for any condition; (5) active systemic infection or skin infection at/near acupuncture sites; and (6) receiving chemotherapy or any drug known to affect xerostomia during the study period. All treatments known to affect salivation had to be stopped 14 days or more prior to enrollment.

### Procedures

Participants were assessed for eligibility and provided informed consent. At baseline, participants received SOH instructions, completed patient-reported outcome forms, and provided sialometry samples. Participants were randomized to 1 of 3 treatment groups: (1) TA, (2) SA, or (3) SOH. Participants in the acupuncture groups had 2 treatments per week for 4 weeks (standard for a course of acupuncture) and received SOH instructions. Patients completed questionnaires and underwent sialometry testing again at week 4; if participants in the TA or SA cohorts had a minor response (10- to 19-point Xerostomia Questionnaire [XQ] decrease from baseline), they continued their assigned acupuncture treatment twice per week for another 4 weeks. Patients who had no response, partial response, or complete response received no further acupuncture treatment and simply completed the remaining assessments. This procedure was used to better reflect acupuncture clinical practice and determine the persistence of improvement without further treatment for those responding. Patients subsequently completed patient-reported outcomes and sialometry testing at weeks 8, 12, and 26. Patients in the SOH group or SA group were offered 3 sessions of TA at no cost post study completion.

### Randomization and Blinding

Patients were randomized in a 1:1:1 ratio to TA, SA, or SOH, using adaptive minimization randomization^23^ stratified by cancer stage, age, sex, time since radiotherapy, mean parotid radiotherapy doses received (left and right side calculated separately, balanced between groups), and baseline XQ scores. To avoid risk of unblinding between TA and SA, we used a previously successful procedure^17^ in which patients were told the study was examining 2 forms of acupuncture compared with a usual care group. As patients in both groups received real needles in real acupoints, this study description is not deception. Participants and site staff were blinded to TA and SA group assignment.

### Acupuncture Treatment

Participants in both groups were placed in a comfortable supine position. A total of 14 points, body and ear, were used for both groups. All sites were applied for 20 minutes.

#### True Acupuncture

Points were selected based on (1) previously published studies reporting xerostomia reversal,^17,18,19,24^ (2) classical traditional Chinese medicine theory,^26,27^ and (3) current understanding of anatomical locations and neurovascular tissues associated with salivary function. Acupuncture points were at 3 sites on each ear (Shenmen, point 0, salivary gland 2-prime^19,20^), a site on the chin (CV24), a site on each forearm (LU7), a site on each hand (LI 1-prime^19,20^), and a site on each leg (K6) with 1 placebo needle at GB32 for a total of 14 sites. For body points, standardized techniques for location were used.

#### Sham Acupuncture

Well-designed clinical acupuncture trials require a sham procedure that is indistinguishable from real treatment, yet inactive. Although no standard has been established for placebo controls in acupuncture trials, nonpenetrating needles placed at inactive points are recommended. A validated, nonpenetrating, telescoping needle with a separate device that attaches it to the skin was used.^28,29^ Although needling anywhere on the ear may cause a physiologic response, prior research has identified 3 nonactive points on the helix of the ear.^30,31,32^

The SA followed the same schedule as TA. Three fixed points were used on each ear located in the middle of the ear helix and are known as helix 2, helix 3, and helix 4.^33^ The sham procedure for body points included sham needles at inactive points: sham location 1: 0.5 cun (a body inch used in traditional Chinese medicine to locate acupoints) below and 0.5 cun lateral to CV 24 on the chin; sham location 2: 0.5 cun radial and 0.5 cun proximal to Sanjiao 6 between Sanjiao and LI channels (bilateral upper extremity); sham location 3: 2 cun above sham location 2 between Sanjiao and LI channels and between LI7 and LI8 (bilateral upper extremity); and sham location 4: 1.0 cun below and 0.5 cun lateral to ST36, between ST and GB channels (bilateral lower extremity) (in traditional Chinese medicine, a cun is an anatomical measure based on the patient’s own body and equals the width of the thumb at the knuckle).^26^ One real acupuncture needle was inserted at GB32, not indicated for dry mouth, to achieve de qi sensation.

#### Needles

The acupuncture needles (Seirin Corp, Kyoto, Japan) used in the ear and LI 1-prime were 40 gauge ×15 mm, and the needles used for all other body points were 36 gauge ×30 mm, conforming to the requirements of the ISO 9002, EN46002, and CE.

Acupuncture was performed by a certified acupuncturist (including M.K.G., J.K.M., and 50 other acupuncturists) designated by each site. Study acupuncturists met state licensing requirements, passed the National Certification Commission for Acupuncture and Oriental Medicine examination (if required by state regulations), and had at least 1 year of clinical acupuncture experience. Group or 1-on-1 training sessions were conducted for each acupuncturist.

#### Standard Oral Hygiene

All patients received SOH information: instructions regarding mouth rinses, lip balms, mild fluoride toothpaste, importance of adequate oral hydration, and other standard advice. All participants continued SOH throughout the study.

### Outcomes

The subjective sensation of dry mouth is not associated with objective saliva flow rate; the US Food and Drug Administration requires patient-reported outcome measures for assessing xerostomia interventions. Patient-reported outcomes including the XQ (primary outcome) and the Functional Assessment of Cancer Therapy–General [FACT-G]; secondary outcome) were completed at baseline and weeks 4, 8, 12, and 26. The Acupuncture Expectancy Scale was collected at baseline for all participants and at week 4 in the TA and SA groups only.

The validated, 8-item XQ is the prime standard measure for xerostomia.^1,2^ Items are summed and transformed linearly to produce a summary score from 0 to 100. Higher scores represent more xerostomia, with a 10-point difference or change considered clinically significant.

The FACT-G quality-of-life instrument is a commonly used questionnaire.^34^ The total score is reported, with higher scores representing better quality of life.

The 4-item Acupuncture Expectancy Scale was used to ensure there were no baseline group differences in expectations of benefit of acupuncture on dry mouth and to assess aspects of blinding after the first 4 weeks of acupuncture for the TA and SA groups.^35^ Higher scores represent greater expectancy that acupuncture will help with dry mouth.

#### Response Assessment

Response was assessed by examining change in XQ scores from baseline to weeks 4, 8, 12, and 26. Response categories included no response (any increase in XQ scores or decrease of <10 points), minor response (10- to 19-point decrease in XQ score), partial response (≥20-point decrease in XQ score), and complete response (complete resolution of xerostomia, with XQ = 0).^1,2^

Adverse events were assessed at each time point using the National Cancer Institute Common Terminology Criteria for Adverse Events, version 5.0.^36^ Only grade 1 to 3 adverse events (as acupuncture is not a life-threatening treatment) that were definitely related, probably related, or possibly related to the intervention were reported.

### Statistical Analysis

Data analysis was performed from March 9, 2022, to May 17, 2023. The primary end point was XQ at week 4. The study was powered to detect a difference of 10 points, accepted as clinically significant on the XQ^1,2^ and on a 0 to 100 scale,^37^ with an assumed SD of 16 between each pair of groups on XQ and required 64 participants per group (N = 192), assuming a *t* test with a 2-sided significance level of *P* = .0167 (adjusting for 3 comparisons) and 84% power. To allow for a dropout rate of up to 20%, 240 patients were recruited.

Primary analyses were 2-sided *t* tests at week 4 comparing all 3 groups. Repeated measures analysis of covariance models were also conducted to estimate differences in each outcome, adjusting for baseline value, group, time, and group by time interaction, assuming an unstructured covariance. Clinical response differences in XQ scores (partial responses or clinically significant xerostomia defined as XQ >30) were examined using χ^2^ tests. Adverse events were summarized by group. All analyses were intention to treat. Secondary sensitivity analyses to account for missing data using multiple imputation for outcomes (XQ, FACT-G, and Acupuncture Expectancy Scale) were performed using monotone regression with 1000 iterations. Age, sex, race and ethnicity, and job status were used for the imputation. Imputation values were restricted to a minimum and maximum range dictated by each outcome. Analyses were performed using SAS, version 9.4 (SAS Institute Inc).^38^

## Results

Of the 258 participants who consented and were randomized, mean (SD) age was 65.0 (9.16) years; median age was 65.2 (IQR, 21.1-84.7) years, 77.9% (n = 201) were men and 22.1% (n = 57) were women, 5.0% (n = 13) were Asian, 6.2% (n = 16) were Hispanic or Latino, and 88.8% (n = 229) were non-Hispanic White (self-reported race and ethnicity), and 64.3% (n = 166) were married. A total of 97.5% (n = 234) had grade 2 xerostomia and were a mean (SD) of 4.21 (3.74) years from diagnosis (range, 1.17-18.42 years); 67.1% (n = 173) had stage IV cancer. Participants were enrolled from 33 NCORP practices over 12 states in the continental US (California, Illinois, Iowa, Michigan, Minnesota, New Mexico, Ohio, Oregon, South Carolina, Tennessee, Texas, and Washington) and Hawaii. There were no significant group differences in demographic or medical characteristics (Table 1 and Table 2).

**Table 1.** Baseline Demographic, Radiation, and Clinical Characteristics by Treatment Group^a^

Characteristic	Participants, No. (%)			
	Combined (N = 258)	TA (n = 86)	SA (n = 86)	SOH (n = 86)
Age at enrollment, mean (SD), y	65.0 (9.2)	65.0 (8.6)	64.4 (9.9)	65.6 (9.0)
Age stratification, y				
<40	2 (0.8)	0	2 (2.3)	0
41-55	43 (16.7)	14 (16.3)	13 (15.1)	16 (18.6)
56-70	143 (55.4)	50 (58.1)	47 (54.7)	46 (53.5)
>70	70 (27.1)	22 (25.6)	24 (27.9)	24 (27.9)
BMI, mean (SD)	26.7 (5.5)	26.0 (4.3)	26.5 (5.7)	27.6 (6.2)
Sex				
Male	201 (77.9)	68 (79.1)	67 (77.9)	66 (76.7)
Female	57 (22.1)	18 (20.9)	19 (22.1)	20 (23.3)
Race and ethnicity^b^				
African American	9 (3.5)	2 (2.3)	5 (5.8)	2 (2.3)
American Indian or Alaska Native	2 (0.8)	1 (1.2)	0	1 (1.2)
Asian	13 (5.0)	4 (4.7)	2 (2.3)	7 (8.1)
Hispanic or Latino	16 (6.2)	3 (3.5)	7 (8.1)	6 (7.0)
Native Hawaiian or Pacific Islander	1 (0.4)	0	0	1 (1.2)
White	229 (88.8)	77 (89.5)	77 (89.5)	75 (87.2)
>1 Race	4 (1.6)	2 (2.3)	2 (2.3)	0
Insurance				
Medicare	101 (39.2)	32 (37.2)	35 (40.7)	34 (39.5)
Medicaid	15 (5.8)	2 (2.3)	5 (5.8)	8 (9.3)
Private	154 (59.7)	56 (65.1)	51 (59.3)	47 (54.7)
None	13 (5.0)	4 (4.7)	5 (5.8)	4 (4.7)
Marital status				
Single (never married)	18 (7.0)	4 (4.7)	10 (11.6)	4 (4.7)
Married/living like married	166 (64.3)	61 (70.9)	48 (55.8)	57 (66.3)
Separated/divorced	34 (13.2)	8 (9.3)	14 (16.3)	12 (13.9)
Widowed	11 (4.3)	4 (4.7)	4 (4.7)	3 (3.5)
Unknown	29 (11.2)	9 (10.4)	10 (11.6)	10 (11.6)
Educational level				
High school	55 (21.3)	18 (20.9)	17 (19.8)	20 (23.3)
Some college/training	88 (34.1)	27 (31.4)	30 (34.9)	31 (36.0)
College degree (BA/BS)	51 (19.8)	20 (23.3)	16 (18.6)	15 (17.5)
Advanced degree	34 (13.2)	11 (12.8)	13 (15.1)	10 (11.6)
Unknown	30 (11.6)	10 (11.6)	10 (11.6)	10 (11.6)
Job status				
Retired	95 (36.8)	37 (43.0)	28 (32.6)	30 (34.9)
Employed (full time/part time)	86 (33.3)	29 (33.7)	26 (30.2)	31 (36.0)
Disabled (unable to work)	22 (8.6)	5 (5.8)	7 (8.1)	10 (11.6)
Other/unknown	55 (21.3)	15 (17.5)	25 (29.1)	15 (17.5)
RTOG scale grade				
Grade 2	234 (97.5)	77 (96.3)	80 (98.8)	77 (97.5)
Grade 3	6 (2.5)	3 (3.8)	1 (1.2)	2 (2.5)
Baseline XQ scores				
<30	10 (3.9)	4 (4.7)	3 (3.5)	3 (3.5)
30-39	19 (7.4)	7 (8.1)	6 (7.0)	6 (7.0)
40-49	37 (14.3)	11 (12.8)	13 (15.1)	13 (15.1)
≥50	192 (74.4)	64 (74.4)	64 (74.4)	64 (74.4)
Baseline AES score	12.1 (4.0)	11.9 (4.1)	11.7 (3.7)	12.7 (4.3)
ECOG score				
0	173 (68.9)	56 (66.7)	61 (72.6)	56 (67.5)
1	75 (29.9)	26 (31.0)	23 (27.4)	26 (31.3)
2	3 (1.2)	2 (2.4)	0	1 (1.2)
Stage of disease				
I	23 (8.9)	8 (9.3)	7 (8.1)	8 (9.3)
II	21 (8.1)	7 (8.1)	8 (9.3)	6 (7.0)
III	41 (15.9)	13 (15.1)	13 (15.1)	15 (17.4)
IV	173 (67.1)	58 (67.4)	58 (67.4)	57 (66.3)
Left mean parotid radiotherapy doses, Gy				
<10	5 (1.9)	1 (1.2)	1 (1.2)	3 (3.5)
10 to <20	25 (9.7)	9 (10.5)	9 (10.5)	7 (8.1)
20 to <30	110 (42.6)	35 (40.7)	38 (44.2)	37 (43.0)
30 to <40	50 (19.4)	17 (19.8)	16 (18.6)	17 (19.8)
40 to <50	21 (8.1)	7 (8.1)	7 (8.1)	7 (8.1)
50 to <60	18 (7.0)	6 (7.0)	6 (7.0)	6 (7.0)
≥60	29 (11.2)	11 (12.8)	9 (10.5)	9 (10.5)
Right mean parotid radiotherapy doses, Gy				
<10	11 (4.3)	3 (3.5)	4 (4.7)	4 (4.7)
10 to <20	26 (10.1)	9 (10.5)	9 (10.5)	8 (9.3)
20 to <30	104 (40.3)	35 (40.7)	34 (39.5)	35 (40.7)
30 to <40	49 (19.0)	17 (19.8)	15 (17.4)	17 (19.8)
40 to <50	24 (9.3)	7 (8.1)	9 (10.5)	8 (9.3)
50 to <60	26 (10.1)	9 (10.5)	8 (9.3)	9 (10.5)
≥60	18 (7.0)	6 (7.0)	7 (8.1)	5 (5.8)

Abbreviations: AES, Acupuncture Expectancy Scale; BA, bachelor of arts; BMI, body mass index (calculated as weight in kilograms divided by height in meters squared); BS, bachelor of science; ECOG, Eastern Cooperative Oncology Group; RTOG, Radiation Therapy Oncology Group; SA, sham acupuncture; SOH, standard oral hygiene; TA, true acupuncture; XQ, Xerostomia Questionnaire.

^a^ There were no significant between-group differences.

^b^ Race and ethnicity were self-reported.

**Table 2.** Least Squares Mean Estimates With 95% CI for Each Treatment Group by Time^a^

Time week	LS mean (95% CI)				Pairwise comparisons					
	TA	SA	SOH	TA v SOH			SA v SOH		TA v SA	
				LS means difference (95% CI)		*P* value	LS means difference (95% CI)	*P* value	LS means difference (95% CI)	*P* value
XQ score										
4	50.59 (47.67 to 53.50)	54.99 (51.95 to 58.03)	57.26 (53.95 to 60.57)	−6.67 (−11.08 to −2.27)		.003	−2.27 (−6.76 to 2.23)	.32	−4.41 (−8.62 to −0.19)	.04
8	48.80 (45.28 to 52.32)	51.20 (47.59 to 54.81)	55.19 (51.30 to 59.08)	−6.39 (−11.64 to −1.15)		.02	−3.99 (−9.30 to 1.31)	.14	−2.40 (−7.44 to 2.64)	.35
12	48.93 (45.29 to 52.57)	49.94 (46.20 to 53.68)	54.81 (50.88 to 58.75)	−5.88 (−11.24 to −0.52)		.03	−4.87 (−10.30 to 0.55)	.08	−1.01 (−6.23 to 4.21)	.70
26	49.22 (45.51 to 52.92)	47.38 (43.58 to 51.18)	54.39 (50.37 to 58.42)	−5.18 (−10.65 to 0.29)		.06	−7.01 (−12.55 to −1.48)	.01	1.84 (−3.47 to 7.14)	.50
FACT-G total										
4	101.59 (99.96 to 103.23)	100.07 (98.39 to 101.75)	97.69 (95.82 to 99.55)	3.91 (1.43 to 6.38)		.002	2.38 (−0.13 to 4.89)	.06	1.53 (−0.82 to 3.87)	.20
8	101.05 (99.14 to 102.96)	100.12 (98.18 to 102.05)	99.51 (97.38 to 101.64)	1.55 (−1.32 to 4.41)		.29	0.61 (−2.27 to 3.49)	.68	0.94 (−1.78 to 3.66)	.50
12	102.05 (100.28 to 103.83)	98.41 (96.60 to 100.23)	97.44 (95.53 to 99.36)	4.61 (1.99 to 7.23)		.001	0.97 (−1.67 to 3.61)	.47	3.64 (1.10 to 6.18)	.005
26	100.45 (98.24 to 102.67)	100.09 (97.82 to 102.35)	98.67 (96.23 to 101.11)	1.78 (−1.52 to 5.08)		.29	1.42 (−1.91 to 4.75)	.40	0.36 (−2.80 to 3.53)	.82
AES score										
4	10.78 (10.03 to 11.52)	10.70 (9.91 to 11.48)	NA	NA			NA		0.08 (−1.00 to 1.16)	.88

Abbreviations: AES, Acupuncture Expectancy Scale; FACT-G, Functional Assessment of Cancer Therapy–General; LS, least squares; NA, not applicable; SA, sham acupuncture; SOH, standard oral hygiene; TA, true acupuncture; XQ, Xerostomia Questionnaire.

^a^ eTable 2 in Supplement 2 provides raw means. Models adjusted for baseline outcome, treatment group, time, and interaction of treatment over time, with an unstructured covariance.

### Enrollment and Retention

**Figure 1.**
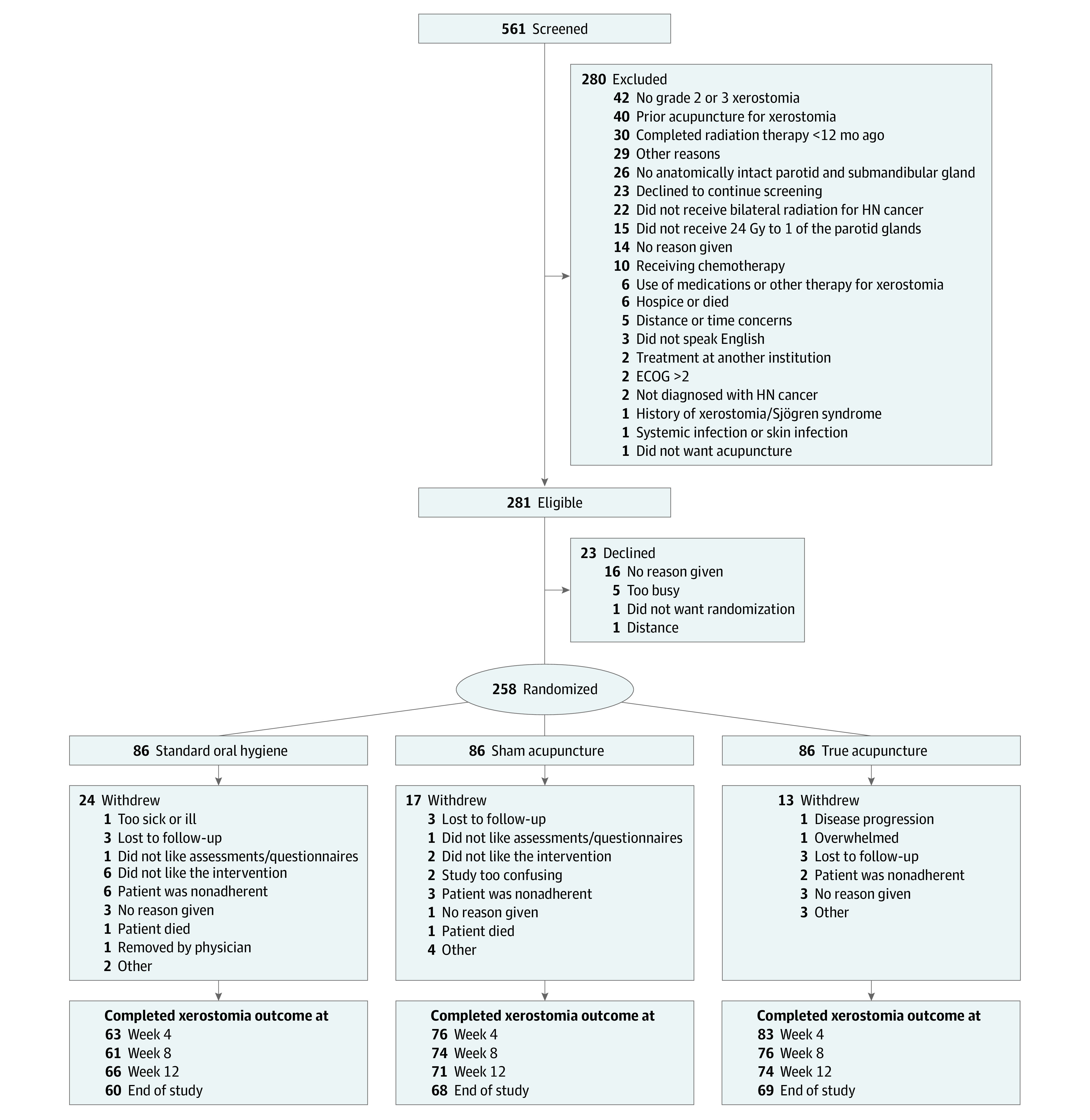
Patient Flowchart ECOG indicates Eastern Cooperative Oncology Group; HN, head and neck.

Of 561 screens, 281 patients (50.1%) were eligible (Figure 1), and 23 (4.1%) declined participation. The remaining 258 participants provided informed consent and then were randomized, resulting in 86 in each group and an 1:1:1 allocation. A total of 54 participants (20.9%) withdrew from the study before the week 4 assessment (TA: 13, SA: 17, and SOH: 24) (Figure 1). There were differential dropout rates at week 4 by group (TA, 2.4%; SA, 10.6%; and SOH, 23.8%; *P* < .001), job status (retired, 6.4%; employed, 10.5%; disabled/unable to work, 27.3%; and other/unknown, 19.2%; *P* = .02), race (African American, 44.4%; American Indian or Alaska Native, 50%; Asian, 7.7%; Native American or Pacific Islander, 100%; White, 10.7%; *P* = .007), and ethnicity (Hispanic, 31.3%; non-Hispanic, 10.9%; *P* = .03).

### Treatments

Acupuncturist experience ranged from 1 to 20 or more years. Adherence was high in the TA and SA groups, with 94.4% (152 patients [TA, 83; SA, 69]) receiving 6 of 8 treatments and 78.3% (126 patients [TA, 72; SA, 54]) receiving all 8 treatments. After the week 4 assessment, 13 patients in the TA group and 12 in the SA group underwent a second course of treatment, with 82% of patients in the TA group and 100% of patients in the SA group receiving 6 or more treatments.

### Xerostomia

There were no significant mean (SD) group differences in XQ at baseline (TA: 63.0 [17.1], SA: 62.1 [17.5], SOH: 63.2 [16.8]; *P* = .91) (eTable 1 in Supplement 2). The analysis of covariance (controlling for baseline XQ) at week 4 revealed a group main effect (*P* = .02), with TA reporting significantly lower XQ scores than SOH (TA: 50.6; SOH: 57.3; difference, −6.67; 95% CI, −11.08 to −2.27; *P* = .003), with marginal differences based on the adjusted *P* value between TA and SA (TA: 50.6; SA: 55.0; difference, −4.41; 95% CI, −8.62 to −0.19; *P* = .04), yet no significant differences between SA and SOH (SA: 55.0; SOH: 57.3, difference, −2.27; 95% CI, −6.76 to 2.23; *P* = .32) (Table 2; eTable 1 in Supplement 2 provides raw means).

**Figure 2.**
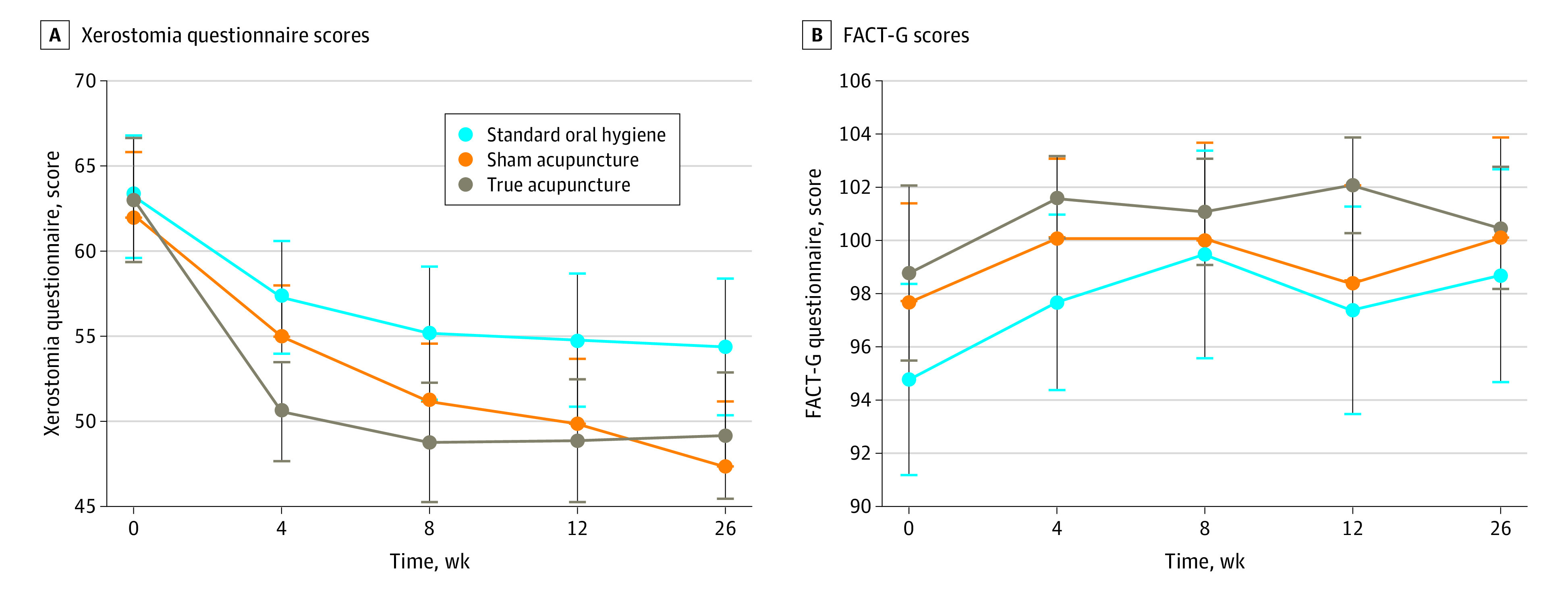
Least Squares Mean Model Estimates for Xerostomia Questionnaire Scores and Functional Assessment of Cancer Therapy–General (FACT-G) Scores by Treatment Group A, Least squares mean model estimates for Xerostomia Questionnaire. Unadjusted baseline mean and 95% CIs presented for reference, but adjusted mean and 95% CIs are presented for weeks 4, 8, 12, and 26. There were statistically significant differences between true acupuncture and standard oral hygiene (*P* = .003) at week 4 and between sham acupuncture and standard oral hygiene at week 26, true acupuncture vs standard oral hygiene at week 8 (*P* = .02), week 12 (*P* = .03), and week 26 (*P* = .06). B, Least squares mean model estimates for FACT-G scores. Unadjusted baseline mean and 95% CIs presented for reference, but adjusted mean and 95% CIs are presented for weeks 4, 8, 12, and 26. There were statistically significant differences between true acupuncture vs standard oral hygiene at week 4 (*P* = .002) and between true acupuncture vs standard oral hygiene (*P* = .001) and true acupuncture vs sham acupuncture (*P* = .005) at week 12.

**Figure 3.**
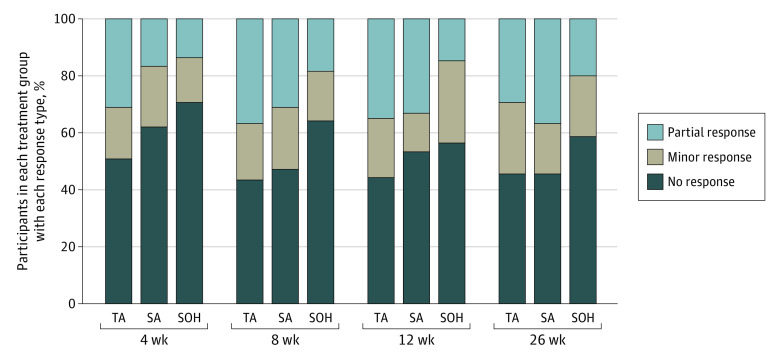
Response Rates Based on Xerostomia Questionnaire (XQ) Scores by Treatment Group at Each Time Point Difference in distributions between true acupuncture (TA) and standard oral hygiene (SOH) was statistically significant at weeks 4, 8, and 12 (*P* = .03 for each time point). Difference in distribution between sham acupuncture (SA) and SOH was significant at week 12 (*P* = .01). Differences were assessed using χ^2^ tests. No response indicates any increase in XQ scores or decrease of less than 10 points; minor response, 10- to 19-point decrease in XQ score; and partial response, 20 points or more decrease in XQ score.

In the mixed-model analyses of repeated measures (Figure 2A), there was a significant group by time interaction revealing group differences at each time point. Similar to the week 4 outcomes, the TA group continued to report statistically significantly lower XQ scores at weeks 8 and 12 than the SOH group, yet by week 26 only the SA group reported lower XQ scores than the SOH group, with no other significant differences. Distributions of response were significantly different between TA and SOH at weeks 4, 8, and 12 (*P* = .03 for each time point) (Figure 3), with twice as many patients in the TA cohort having a partial response at week 4 (TA: 31.3% [26 of 83]; SA: 17.1% [13 of 76]; SOH: 14.1% [9 of 64]). Similarly, proportions of participants with clinically significant xerostomia (defined as XQ >30) were significantly different for TA compared with SOH at weeks 4 (84.3% [70 of 83] vs 95.3% [61 of 64]; *P* = .03) and 12 (73.0% [54 of 74] vs 90.9% [60 of 66]; *P* = .01). No other differences were observed for clinically significant xerostomia rates.

### FACT-G

The mixed-model repeated-measures analyses for the FACT-G scores followed a similar pattern as XQ, revealing significant group differences at week 4 with higher scores in the TA compared with the SOH cohort (TA: 101.6; SOH: 97.7; difference, 3.91; 95% CI, 1.43-6.38; *P* = .002), and week 12, with higher scores in the TA vs SA cohort (TA: 102.1; SA: 98.4; difference, 3.64; 95% CI, 1.10-6.18; *P* = .005) and higher scores in the TA compared with the SOH cohort (TA: 102.1; SOH: 97.4; difference, 4.61; 95% CI, 1.99-7.23; *P* = .001), with no between-group differences at week 26 (Table 2 and Figure 2B). Multiple imputation sensitivity analyses were robust to the results found in the primary analyses, with the exception of TA vs SA at week 4 (eTable 2 in Supplement 2).

### Acupuncture Expectancy Questionnaire

There were no significant group differences in expectations of the effect of acupuncture on dry mouth symptoms at any time point. This suggests that blinding was maintained (Table 1 baseline and Table 2, week 4).

### Adverse Events

No serious adverse events occurred. Six adverse events were reported across all groups (SOH: 0; SA: 3; TA: 3). Adverse events with SA included 1 patient, facial edema, 1 patient, flulike symptom, and 1 patient, joint pain (all grade 3). Adverse events with TA included 1 patient, hypertension (grade 3), 1 patient, headache (grade 1), and 1 patient, bruising (grade 1).

## Discussion

To our knowledge, this study is the first randomized, sham-controlled, phase 3, multicenter clinical trial to evaluate acupuncture for chronic RIX in patients with head and neck cancer after radiotherapy. The results are consistent with several past smaller trials.^12,24,39,40,41,42,43^ Although there were no statistically significant differences between TA and SA based on the adjusted *P* values, there was a 4.4-point difference (*P* = .04) with the only statistically significant differences emerging between TA and SOH. Similarly, 31.3% of patients receiving TA had a partial response at week 4 vs only 17.1% with SA and 14.1% with SOH, suggesting a clinically significant benefit with TA. Differences between TA and SOH remained through week 12. The SA group improved over time and reached statistically significant differences from SOH by week 26, but there were no significant differences between TA and SA at week 26, suggesting that both forms of acupuncture were effective. Although the absolute differences between groups did not reach 10 points on the XQ, the proportion of patients no longer meeting the clinical definition for xerostomia was only improved with TA vs SOH. Moreover, examination of changes in quality of life showed improvement with TA compared with SOH at week 4; the differences reached statistical significance between TA and SA and between TA and SOH at week 12. Both XQ and quality-of-life findings suggest that perhaps subsequent maintenance acupuncture treatments are needed to maintain or enhance the effects of acupuncture.

The sham procedure was effective in maintaining blinding, with both groups reporting high expectations of benefit after week 4. There were differential dropout rates at week 4, mainly noted in patients in the SOH group. Because real needles were inserted in the helix of the ears and a real point on the leg, the sham treatment cannot be considered a true placebo. Although placebo-controlled trials impart important information toward understanding mechanisms, the choice of placebo comparators in acupuncture trials remains highly debated. Thus, as other large, 3-arm acupuncture trials have demonstrated, the most relevant comparison is between TA and usual care.^44,45,46^

Although acupuncture mechanisms are not well understood, findings from multiple studies suggest possible central nervous system effects through manipulation of the fascia.^15,16,47,48,49^ Studies have revealed significant increased blood flow in the skin of the cheek of patients with xerostomia.^47^ Other plausible hypotheses suggest that increased production of certain neuropeptides after acupuncture stimulation may cause vasodilation and increased microcirculation.^15,16,48^ Deng et al^49^ explored neuronal substrates during acupuncture for RIX using functional magnetic resonance imaging. True acupuncture was associated with activation of areas of the brain where sensory stimuli and expectation/suggestion signals are integrated, an effect not seen with SA. Furthermore, TA caused significantly more saliva production, also related to neurologic changes.

### Strengths and Limitations

This study had a number of strengths and limitations. The trial was unable to recruit a large sample of racially and ethnically underserved participants. Yet it recruited participants from more than 30 clinics, representing one of the largest acupuncture studies, increasing generalizability. Some positive effects associated with acupuncture may be due to nonspecific factors (eg, conditioning, expectations, self-empowerment, patient-practitioner relationship) that were not assessed.^50^ Most patients only received 4 weeks of acupuncture, and no maintenance treatments were provided.

## Conclusions

Acupuncture is minimally invasive and inexpensive, has a low incidence of adverse effects, and was found to be superior to standard care in treating chronic RIX after treatment in this large phase 3 trial. Based on these and other findings, acupuncture may be considered as a treatment option for patients with chronic RIX.

## Notes

Supplement 1.
Trial Protocol

Supplement 2. **eTable 1.** Raw Means and Standard Deviations for All Outcomes Listed by Treatment Group and Time **eTable 2.** Multiple Imputation Sensitivity Analysis for Least Square Mean Estimates With 95% Confidence Intervals for Each Treatment Group by Time

Supplement 3.
Data Sharing Statement
