# Supplementary material for: Acupuncture for Chronic Radiation-Induced Xerostomia in Head and Neck Cancer: A Multicenter Randomized Clinical Trial
Source: JAMA Netw Open. 2024 May 13;7(5):e2410421. doi: 10.1001/jamanetworkopen.2024.10421 (PMC11091764; doi:10.1001/jamanetworkopen.2024.10421)
Supplement: Supplement 1. — Trial Protocol [file jamanetwopen-e2410421-s001.pdf]

CI Protocol #: WF 97115
WFU Protocol #: 97115
IND# (if applicable): NA

PROTOCOL TITLE: A Phase III Prospective Randomized Trial of Acupuncture for Treatment of Radiation-Induced Xerostomia in Patients with Head and Neck Cancer

Consortium Name: **Wake Forest NCORP Research Base**
Name of Consortium Multi-Principal Investigator: **Glenn Lesser, MD**
Wake Forest School of Medicine
Department of Internal Medicine
Section on Hematology and Oncology
Medical Center Boulevard
Winston-Salem, NC 27157
Telephone (336) 716-0891
Fax: (336) 716-6275
Email: glesser@wakehealth.edu

Consortium Name: **Wake Forest NCORP Research Base**
Name of Consortium Multi-Principal Investigator: **Kathryn Weaver, PhD, MPH**
Associate Professor of Public Health Sciences
Department of Social Sciences & Health Policy
Medical Center Boulevard
Winston-Salem, NC 27157
Telephone: (336) 713-5062
Fax: (336) 716-7554
Email: keweaver@wakehealth.edu

Organization Name: Wake Forest School of Medicine
Protocol Principal Investigator: Suzanne C. Danhauer, PhD
Department of Social Sciences & Health Policy
Division of Public Health Sciences
Medical Center Boulevard
Winston-Salem, NC 27157
Telephone (336) 716-7402
Fax (336) 716-7554
Email: danhauer@wakehealth.edu

Coordinating Center: Wake Forest NCORP Research Base
Department of Social Sciences & Health Policy
Medical Center Boulevard, 525 @ Vine 4th Floor (Box 573152)
Winston-Salem, NC 27157
Telephone: (336) 716-0891
Fax: (336) 716-6275
Email: resbase@wakehealth.edu

Protocol Chairman: Lorenzo Cohen, PhD
The University of Texas MD Anderson Cancer Center
1400 Holcombe Blvd., Unit 0462
Houston, Texas 77030-4009
PH: (713) 745-4260
FAX: (713) 745-2437
Email: lcohen@mdanderson.org

Protocol Co-Chair:

Mark Chambers, DMD, MS
The University of Texas MD Anderson Cancer Center 1515 Holcombe Blvd., Unit 1445
Houston, Texas 77030-4009
PH: (713) 745-2672
FAX: (713) 794-4662
Email: mchamber@mdanderson.org

Collaborators:

M. Kay Garcia, DrPH, MSN, RN, LAc
The University of Texas MD Anderson Cancer Center 1400 Holcombe Blvd., Unit 462
Houston, Texas 77030-4009
PH: (713) 563-7413
FAX: (713) 745-2437
Email: kaygarcia@mdanderson.org

Joseph S. Chiang, MD
The University of Texas MD Anderson Cancer Center 1400 Holcombe Blvd., Unit 0409
Houston, Texas 77030-4009
PH: (713) 794-5363
FAX: (713) 563-9335
Email: jchiang@mdanderson.org

David Rosenthal, MD
The University of Texas MD Anderson Cancer Center 1515 Holcombe Blvd., Unit 0097
Houston, Texas 77030-4009
PH: (713) 563-2300
FAX: (713) 563-2366
Email: dirosenthal@mdanderson.org

Peiying Yang, PhD
The University of Texas MD Anderson Cancer Center 1400 Holcombe Blvd., Unit 0462
Houston, Texas 77030-4009
PH: (713) 563-7531
FAX: (713) 745-2437
Email: pyang@mdanderson.org

Statistician:

Emily V. Dressler, PhD
Department of Public Health Sciences
Department of Biostatistics and Data Science
Wake Forest University School of Medicine
Medical Center Boulevard
Winston-Salem, NC 27157
PH: (336) 713-0917
FAX: (336) 716-6427
Email: edressle@wakehealth.edu

Participating Organizations

WAKE/ Wake Forest NCORP Research Base

Grant: 5UG1CA189824 (NCI)
5R01CA148707 (NCI)

Agent(s)/Supplier: ***N/A***
Protocol Amendment #: 14
Protocol Version Date: 12/26/2019

Amendments Numbers & IRB Approval Dates:
Amend 2: 08/13/15 (Initial)
Amend 3: 02/11/16 (Personnel Change)
Amend 4: 02/11/16 (WF Change Only)
Amend 5: Withdrawn
Amend 6: 03/03/16 (WF Change Only)
Amend 7: 03/08/16 (Personnel Change)
Amend 8: 05/12/16 (Personnel Change)
Amend 9: 05/15/16
Amend 10: 04/26/17
Amend 11: 05/01/18
Amend 12: 07/01/19
Amend 13: 10/17/2019
Amend 14: [enter IRB approval date prior to distributing to sites]

ACUPUNCTURE – SCHEMA

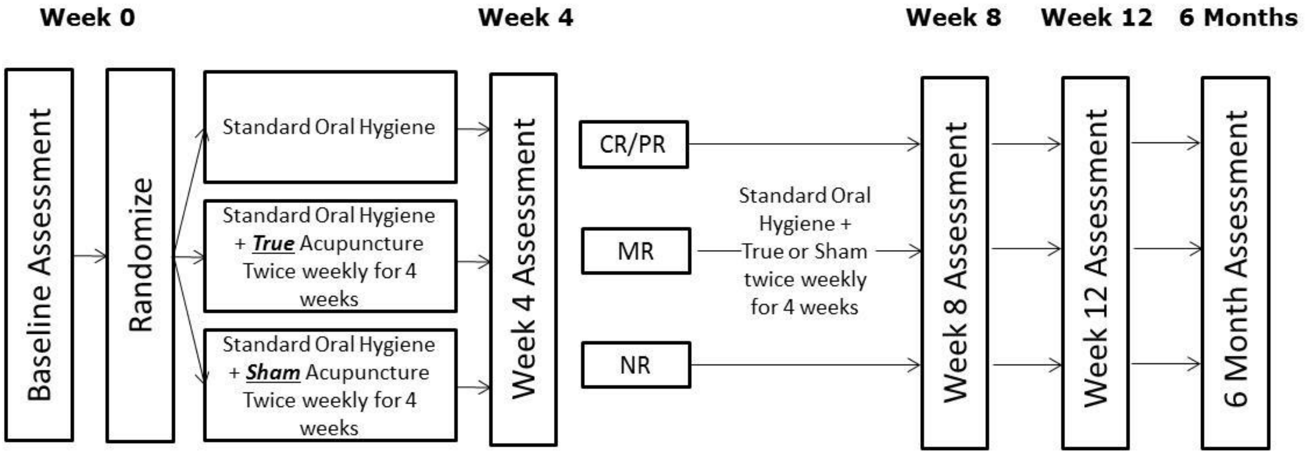

Accrual Goal: 240

TABLE OF CONTENTS

1.0 OBJECTIVES 7
1.1 Primary Objective 7
1.2 Secondary Objective 7
2.0 BACKGROUND 7
2.1 Rationale 7
3.0 PATIENT SELECTION 11
3.1 Inclusion Criteria 11
3.2 Exclusion Criteria 12
3.3 Study Population 12
4.0 STUDY DESIGN 12
4.1 Enrollment Procedures 12
4.2 Stratification/Randomization Procedures 12
5.0 TREATMENT PLAN 13
5.1 Acupuncture 13
5.2 Sham Treatment 15
5.3 Standard of Care 16
5.4 Needling/Training Procedures 16
6.0 STUDY REQUIREMENTS 17
6.1 Study Plan 17
6.2 Baseline Assessments 18
6.3 On-Study Assessments 18
6.4 Off-Study Assessments 19
6.5 Study Compliance 19
6.6 Intervention Delay 19
6.7 Study Follow-Up 20
7.0 MEASUREMENT OF EFFECT 20
7.1 Xerostomia Questionnaire (XQ) 20
7.2 MD Anderson Symptom Inventory for Head and Neck Cancer (MDASI-HN) 20
7.3 The Functional Assessment of Cancer Therapy (FACT-G) 20
7.4 Acupuncture Expectancy Scale (AES) 20
7.5 Study Calendar of Events 22
8.0 SIALOMETRY 23
8.1 Saliva Flow Examination 23
8.2 Saliva Constituent Analyses 23
9.0 ADVERSE EVENTS AND REPORTING REQUIREMENTS 23
9.1 Protocol Specific Reporting for Adverse Events (AEs) 24
9.2 Protocol Specific Reporting for Serious Adverse Events (SAEs) 24
9.3 Guidelines to Determine Grade and Severity of AEs and/or SAEs 25
9.4 Follow-Up of SAEs 25
10.0 STASTICAL CONSIDERATIONS 26
11.0 STUDY ACCRUAL 27
12.0 DATA MANAGEMENT 27
13.0 DATA MONITORING COMMITTEE 28
14.0 SITE REGISTRATION 28
15.0 REFERENCES 30

Consent

Appendix 1 - Data Submission Checklist
Appendix 2 - Eligibility Checklist/Enrollment Form
Appendix 3 - FACT-G
Appendix 4 - MDASI-HN
Appendix 5 - Xerostomia Questionnaire
Appendix 6 - Flow Sheet/Addenda
Appendix 7 - Acupuncture Expectancy Scale
Appendix 8 - Oral Hygiene Instructions
Appendix 9 - Screening Log
Appendix 10 - Sialometry
Appendix 11 - Toxicity/Adverse Events Report Form
Appendix 12 - Concomitant Medications
Appendix 13 - TAT
Appendix 14 - SAT
Appendix 15 - Patient Status Form
Appendix 16 - Stored Saliva Sample Transmittal Form

1.0 OBJECTIVES

1.1 Primary Objective

1. To determine whether or not acupuncture can symptomatically improve moderate or severe xerostomia (grade 2 or 3) due to head/neck radiotherapy.

1.2 Secondary Objective

1. To explore the duration of response (up to a maximum of 6 months) in the subgroup of patients who report a response to the acupuncture intervention.
2. Examine group differences in saliva flow using unstimulated and stimulated whole salivary flow rates.
3. Examine whether true acupuncture results in better overall QOL than sham acupuncture or standard of care.
4. Determine the effects of acupuncture on saliva-based factors including pH, buffering capacity, and viscosity as well as levels of total protein, calcitonin-gene related peptide, and vasoactive intestinal polypeptide.
5. Examine the role of expectancy for the benefits of acupuncture in predicting outcomes. The role of expectancy as a moderator of the effects of treatment will be thoroughly examined.

2.0 BACKGROUND

2.1 Rationale

Xerostomia is a major quality-of-life (QOL) limiting problem among cancer patients who have received radiation treatment to the head and neck area. The impact of xerostomia on QOL among cancer patients is well established[1-4]. Patients suffer from a decreased or total lack of saliva secretion leading to the subjective discomfort of dry mouth as well as other clinical problems such as difficulty speaking, eating, swallowing, taste aberration, insufficient nutritional intake, weight loss, ulceration, mucositis, pain, fungal infection, gingivitis and tooth structure deterioration. A recent study by Lin and colleagues[5] found quality of life scores were significantly correlated with xerostomia scores.

It is estimated more than 40,000 patients are diagnosed annually with head and neck cancer[6]. Approximately 80% will receive radiotherapy and xerostomia is a common sequela of treatment [7]. The pathology and course of xerostomia is not well described, and several approaches to treatment, including saliva substitutes, chewing gum, lozenges, pilocarpine, nicotinamide, and amifostine, have been attempted with limited results. Thus far, the few available therapies have demonstrated a low success rate or low acceptance by patients due to unpleasant side effects. Currently, the most widely used treatment is orally administered pilocarpine hydrochloride. The limited positive response is short lived and the cholinomimetic effect such as sweating, rhinitis, headaches, nausea and urinary frequency significantly limit its application. In addition to pharmaceutical stimulation, many mechanical devices have been offered ranging from electrical stimulation to the tongue and palate to the extreme hyperbaric oxygen therapy. Each has limited and short-lasting results[8].

Several encouraging reports have been published using acupuncture to stimulate saliva flow among radiation induced xerostomia patients [9-15]. These studies were conducted in different countries, by different investigators, using different acupuncture points, yet all produced similar positive results. One study demonstrated long-term effect (>3 years) of saliva production[11]. An additional study revealed a significant

increase in the blood flux in the skin of the cheek of xerostomia patients [16]. This increase in blood flow may affect the metabolism of the salivary gland causing increased salivary secretion. Other plausible hypotheses suggest increase production of certain neuropeptides after acupuncture stimulation that may cause vasodilation and an increase of microcirculation[13,14,17].

A recently completed, non-randomized, single-arm pilot study [18] at MD Anderson assessed whether radiation-induced xerostomia could be reversed using acupuncture. Quantification of response was measured both subjectively and objectively. Subjective measures based on self-assessment of individual symptoms associated with dry mouth were collected using the Xerostomia Inventory (XI) and the Patient Benefit Questionnaire (PBQ). Objective measures were performed using both unstimulated whole salivary flow rates (UWSFRs) and stimulated salivary flow rates (SSFRs). A total of 20 patients received 8 acupuncture treatments over 4 weeks with follow-up assessments at week 5 and week 8 (1-month).

Tables 1 & 2 provide results from both subjective measures. For the XI (high score = increased severity of xerostomia), differences between baseline and end of acupuncture treatment (week 4) and 1 month follow-up (week 8) were highly statistically significant (p = 0.0004 and p = 0.0001, respectively). Mean scores declined from 36.3 at baseline to 31.6 and 29.9 at weeks 4 and 8 respectively. For the PBQ (low score = increased severity of xerostomia), differences between baseline and end of acupuncture treatment (week 4) and 1 month follow-up (week 8) were also very highly significant (p = 0.0004 and p = 0.0011, respectively). Mean PBQ scores increased from 43.94 at baseline to 56.85 at week 4 and 56.10 at 1-month. In Tables 1 and 2 the standard deviations presented are for the single time point measures and the p values were obtained from Wilcoxon signed-rank test, which used the change from baseline for each individual as the outcome measure.

Table 1. XI scores compared to baseline

Week	Mean	SD	P value
Baseline	36.3	7.71	---
Week 1	36.0	7.01	0.4708
Week 2	34.1	7.27	0.0067
Week 3	32.9	7.32	0.0187
Week 4	31.6	6.66	0.0004
Week 5	31.4	6.80	0.0007
Week 8 (1-month)	29.9	7.96	0.0001

Table 2. PBQ scores compared to baseline

Week	Mean	SD	P value
Baseline	43.94	12.93	---
Week 1	47.10	14.33	0.0675
Week 2	50.10	13.42	0.0029
Week 3	53.60	13.11	0.0010
Week 4	56.85	13.22	0.0004
Week 5	57.78	14.53	<0.0001
Week 8 (1-month)	56.10	12.92	0.0011

For objective measures, no significant differences were found for either UWSFRs or SSFRs; however, mean saliva output did increase from baseline to end of follow-up at 1 month. For UWSFRs, mean saliva at baseline was 0.67g and increased slightly to 0.77g at 1-month, with the greatest difference occurring at week 1 (0.99g, p=0.08). For SSFRs, mean saliva output increased from 1.92g at baseline to 1.94g at week 8, with the most change also occurring at week 1 (2.59g, p=0.13). Tables 3 & 4 provide sialometry results.

Table 3. UWSFR compared to baseline

Week	Mean	SD	P value
Baseline	0.67	0.62	---
Week 1	0.99	0.88	0.08
Week 2	0.71	0.73	0.65
Week 3	0.87	0.88	0.27
Week 4	0.79	0.79	0.54
Week 5	0.68	0.72	0.57
Week 8 (1-month)	0.77	0.81	0.46

Table 4. SSFR compared to baseline

Week	Mean	SD	P value
Baseline	1.92	1.70	---
Week 1	2.59	2.29	0.13
Week 2	2.24	1.86	0.26
Week 3	2.01	1.55	0.40
Week 4	2.12	1.48	0.26
Week 5	2.08	1.46	0.21
Week 8 (1-month)	1.94	1.38	0.62

Both basal and stimulated salivary flow rates vary significantly among individuals. UWSFRs have been reported to range from 0.08 to 1.83 mL/min, a difference of more than 20 fold. SSFRs vary even more, from 0.2 to 5.7 mL/min. Within this wide range, subjective perception of dry mouth and objective signs of salivary gland dysfunction do not always correlate. Subjective feelings of oral dryness (xerostomia) are not reliable indicators of flow rate. Impaired salivary gland function can exist without xerostomia, which can, conversely, exist with normal salivary gland function. Thus, difficulty lies in the fact that there is no definitive threshold of increased saliva output that results in a clear clinical benefit.

Because the FDA uses subjective outcomes as the standard for drug approval in treating xerostomia, summed XI scores were used to determine clinical significance. A partial response (PR) was defined as a difference on the XI $\geq$ 6 points from baseline or 30% of starting score if baseline <20; however, no patients in this population had a baseline score < 20 points. A minor response (MR) was defined as a difference < 6 points but at least 3 points from baseline, and no response (NR) was defined as the absence of either PR or MR.

The overall expected response rate was 40%. At the end of acupuncture treatment on week 4, 44.44% of participants achieved a PR, and on week 8, 55.56% had achieved a PR. These results are provided in Table 5.

Table 5. Clinical significance as determined by XI scores

Week	NR (n / %)	MR (n / %)	PR (n / %)
Week 1	13 / 68.42	5 / 26.32	1 / 5.26
Week 2	10 / 58.82	4 / 23.53	3 / 17.65
Week 3	7 / 38.89	5 / 27.78	6 / 33.33
Week 4	5 / 27.78	5 / 27.78	8 / 44.44
Week 5	6 / 33.33	2 / 11.11	10 / 55.56
Week 8	3 / 16.67	5 / 27.78	10 / 55.56

This small pilot study is viewed as a positive trial. However, since completion of the trial, a new instrument has become available that covers questions from both the XI and the PBQ. Thus, for the proposed study, we will use the Xerostomia Questionnaire (XQ) as the subjective measure in place of the XI and the PBQ. This will reduce the time burden placed on patients while providing the same information. The XQ questionnaire has been validated in patients with head and neck cancer [7,19].

Recently, we also completed a randomized xerostomia prevention trial in Shanghai, China where patients were randomly assigned to undergo acupuncture (n = 39) or usual care (n = 46) throughout their 7 weeks of radiotherapy. Similar, but not identical, acupuncture points were used. There where statistically and clinically significant group differences in subjective outcomes (general QOL and xerostomia) and saliva flow rates starting as early as 3 weeks into the 7 weeks of radiotherapy. The group differences remained 1 and 6 months after the end of radiotherapy. The placebo procedure has also been tested in a small pilot trial conducted in Shanghai, China. Twenty patients undergoing radiotherapy to the head and neck were randomized to either real or sham acupuncture. Patients were asked at week 4 and at the end of week 7 whether they thought they were in the true or sham group or uncertain and whether they thought the acupuncture was helpful for their dry mouth (yes/no). All patients thought they were in the true acupuncture group, and all thought the treatment was useful for their dry mouth. Group differences did not reach statistical significance (which is not surprising due to the small sample size), but differences in XQ scores by week 4 were greater than 10 points, suggesting the differences were clinically significant [week 4 mean (SD): true = 23.6 (8.8) vs sham =
36.1 (13.2)]. Importantly, the means and standard deviations were similar to those in the previous larger completed study that compared acupuncture to standard care.

Finally, in the proposed trial, we will evaluate participant expectations on outcomes. This will allow further exploration of how psychological phenomena may be related to outcomes in acupuncture research. Although well-conducted, placebo-controlled clinical trials have offered insights into the efficacy of acupuncture, the substantial effect seen in many placebo acupuncture groups presents a significant challenge in interpreting treatment results. Many studies, including several meta-analyses[20-26], have found that both real and placebo acupuncture produce statistically and clinically significant changes when compared to no treatment or standard/enhanced medical care. These findings suggest that the effect found in placebo acupuncture groups cannot be entirely attributed to either regression to the mean or natural disease

processes; thus, the “non-specific” effect of acupuncture must be responsible in part for patients’ clinical response. Nevertheless, until we can parse the components that make up the “non-specific” effect, this effect, powerful as it may be, is likely to be discarded from a scientific standpoint. Years of psychological research has found that expectancy is one of the central pieces in this so-called “non-specific” effect. In particular, response expectancy, a form of outcome expectancy defined as “expectations held by the individual about one’s own emotional and physiological response” related to a situation or therapy, may produce important clinical changes[28]. In a systematic review, Crow et al.[28] showed that when clinicians stated positive outcome expectations as opposed to uncertain expectations, most studies found improvements in patient self-reports of anxiety, pain, and distress.

A few studies have explored the association between expectancy and clinical outcomes in the context of acupuncture[29,30]. While these studies provided preliminary evidence that greater expectancy may produce better clinical response, none used a validated instrument to measure expectancy. In this study, we will use an Acupuncture Expectancy questionnaire that has been validated in both English and Mandarin[31]. We will also use the MD Anderson Symptom Inventory for Head and Neck Cancer (MDASI-HN) and the Functional Assessment of Cancer Therapy (FACT-G) questionnaires to collect quality of life information.

Investigating expectancy and quality of life as part of the complex mind-body interactions of acupuncture care will likely yield important knowledge that is both scientifically sound and clinically meaningful to patients who suffer from xerostomia and other distressing symptoms.

3.0 PATIENT SELECTION

3.1 Inclusion Criteria

1. Must be at least 18 years of age and able to give informed consent.
2. Must be able to read, write and understand English.
3. Must have a diagnosis of head/neck cancer.
4. Must have received bilateral radiation therapy, and subsequently developed grade 2 or 3 xerostomia, according to modified RTOG scale:

   - Grade 0 – None
   - Grade 1 – Slight dryness of mouth (good response on stimulation and no significant dietary alterations necessary)
   - Grade 2 – Moderate dryness of mouth (poor response on stimulation and altered oral intake required such as frequent water, oral lubricants, or soft-moist foods)
   - Grade 3 – Complete dryness of mouth (no response on stimulation and difficult oral alimentation; IV fluids, pureed diet or tube feedings may be required)
   - Grade 4 – Fibrosis

5. Must have received external beam radiation with curative intent.
6. Must have completed radiotherapy at least 12 months prior to entry.
7. Must have anatomically intact parotid glands and at least one submandibular gland. A focused (head/neck) history and exam conducted by a physician or dentist within the past year is required.
8. Have never had acupuncture for xerostomia.
9. Must have ECOG performance status of 0-2.

3.2 Exclusion Criteria

1. History of xerostomia, Sjogren's disease or other illness known to affect salivation prior to head/neck radiation.
2. Suspected or known closure of salivary gland ducts on either side. (Patients addressed in inclusion criteria 7 (i.e. those who have had one submandibular gland removed) are expected to have closure of the duct to the removed submandibular gland and will be exempt from this exclusion criteria.)
3. Currently receiving or planning to receive other xerostomia treatment, including drugs, herbs or devices. All other treatments known to affect salivation should be stopped at least 14 days prior to enrollment.
4. Have received any investigational new drug within the past 30 days or planning to receive such during the study period.
5. Active systemic infection or skin infection at or near the acupuncture sites.
6. Receiving chemotherapy during study period.

3.3 Study Population

This study will enroll both male and female patients who have received radiation for the treatment of head and neck cancer. A study recruitment letter is located on the WF NCORP Research Base website for submission to your IRB for approval should the site decide to use as a mail out for recruitment purposes.

The MD Anderson Integrative Medicine Program's recommended safety guidelines for patients receiving acupuncture are provided as a helpful guide on the WF NCORP Research Base website.

4.0 STUDY DESIGN

4.1 Enrollment Procedure

Eligible and consented patients will be enrolled only after the enrolling institution submits a completed IRB approval letter to the Wake Forest NCORP Research Base. IRB approval will be verified. Patients must meet all inclusion/exclusion criteria to be eligible for the protocol. Any patient who does not satisfy all eligibility requirements cannot be entered onto the protocol. Screening logs (Appendix 9) should be kept on all patients screened (approached, accrued, failed) for this trial. Logs should be made readily available for review and data collection if requested.

Once the patient's eligibility is confirmed and the patient has been consented, the baseline assessments should be completed (see Section 6.2). Then the patient can be enrolled to the study using the WFNCORP RB website enrollment system. A unique case number and blinded study arm assignment will be given to each patient. A confirmation of patient enrollment will be generated and conveyed to the enrolling institution for verification purposes. If the patient is randomized to an acupuncture arm, the Research Base will relay the arm assignment to the participating acupuncturist(s) for that site.

4.2 Stratification/Randomization Procedure

Patients who have met all eligibility criteria will be randomized at the time of enrollment to standard oral hygiene, standard oral hygiene + true acupuncture twice weekly for 4 weeks, or standard oral hygiene + sham acupuncture twice weekly for 4 weeks by a form of adaptive randomization, called minimization, because simple randomization could result in covariate imbalances [33]. Statistical adjustment of covariates can take imbalances into consideration, but results are generally more credible when they are obtained from groups with comparable baseline distributions. In minimization, group assignment is done

sequentially. Before a participant is assigned to a group, the number of already randomized participants with similar covariate characteristics is totaled. The totals are computed based on marginal sums so that each covariate is considered separately. The treatment assignment for a participant is then based on which treatment group assignment would produce the best overall balance with respect to the covariate characteristics. Minimization is similar to stratification in that participant characteristics are used to assign participants to the treatment conditions. Minimization, however, results in better group balance and does not suffer from the limitations of stratification, especially when several participant factors are used. In this study, the patient characteristics used for group assignment will be stage of disease (for staging questions please contact the Research Base for assistance), age, sex, time since the end of radiotherapy, the mean parotid radiotherapy doses received (left and right side calculated separately and balanced between groups), and baseline XQ scores. Randomization will be conducted by using a centralized website to ensure equal distribution across all groups and balance at each site. All sites and study staff and patients will be blinded to randomization of the acupuncture arms. At the time of enrollment, the site will be automatically notified of blinded group assignment by the Research Base. The Research Base will provide acupuncturists with unblinded group information.

5.0 TREATMENT PLAN

5.1 Acupuncture

The acupuncture points will be at three sites on each ear (Shenmen, Point Zero, Salivary Gland 2-prime), a site on the chin (CV24), a site on each forearm (Lu7), a site on each hand (LI 1-prime), a site on each leg (K6), and one placebo needle at Gb32 for a total of 14 sites. All sites will be applied for 20 minutes. For body points, standardized techniques for location will be utilized, which are based on anatomical landmarks as well as proportional measurements using the patient's own body. For example, finger breadth is based on each patient's middle finger, and the proportional unit of measure, the "cun," is defined as the distance between the two medial ends of the creases of the interphalangeal joints when the middle finger is flexed[34]. Earpoint locations will mimic standard practice and be identified by the acupuncturists.

The specific acupoints to be used were selected based on: 1) previous published studies [4,9,35] showing successful results of reversing xerostomia; 2) indications according to the classical theory of Traditional Chinese Medicine (TCM)[34,36], and; 3) current understanding of the various anatomical locations and neurovascular tissues. Although many point combinations could be used, the investigators have attempted to identify a set of acupoints that integrates TCM and biomedicine. The sites are summarized below:

CV 24: Above the chin, in the depression in the centre of the mentolabial groove (approximately midway between the chin and lower lip). This point is supplied by branches of the inferior labial artery and vein and the mental nerve.

The Chinese name for this point is *Chengjiang* (translated as "container of fluids.") It is a point where 3 other channels meet (Du, LI, and St). The CV channel winds around the mouth and terminates under the eyes. According to TCM theory, body fluids are considered Yin. Thus, xerostomia is a form of Yin deficiency, and the CV channel is one of the most important channels for tonifying and nourishing Yin.

Although this point has not been closely investigated for the treatment of xerostomia, several clinicians and renowned researchers in this area recommend its inclusion based on their clinical experience.

Lu 7: This point is located superior to the styloid process of the radius, 1.5 cun above the transverse crease of the wrist. If the index finger is placed in the anatomical "snuffbox" located at the radial aspect of the wrist (i.e., LI 5) and moved proximally over the styloid process of the radius, the point is located in the depression in the cleft between the tendons of brachioradialis and abductor pollicis longus, 1.5 cun proximal to the transverse wrist crease. This point is supplied by the cephalic vein and branches of the radial artery and vein. It is innervated by the lateral antebrachial cutaneous nerve and the superficial ramus of the radial nerve.

In TCM, Lu 7 is known as the *Gao Wu* command point for the head and neck area. The lungs in TCM are also responsible for water metabolism, and the lung channel ascends to the throat. Lu 7 is a paired confluent point with K6 and indicated for dry throat. For this function, it is needled proximally towards the elbow. It is also considered a confluent point of the conception vessel (CV) and has the additional function of supporting CV 24 in this study.

K 6: This point is located in the depression 1 cun below the prominence of the medial malleolus in the groove formed by the 2 ligamentous bundles. It is supplied by the posterior tibial artery and vein. It is innervated by the medial crural cutaneous nerve and the deeper tibial nerve.

In TCM, K 6 is known as the "shining sea" point. When paired with its confluent point Lu 7, one of its primary functions is to nourish yin and clear heat from the throat. In addition, by activating 2 channels (i.e., Kidney channel and Yin Motility channel), K 6 is a main distal point for treating Yin deficiency and dryness of the throat.

LI 1-prime: This point is located slightly medial and distal to the traditional *jing well* point, LI 1. It is on the radial side of the index finger, at the edge of the nail and approximately 0.1 cun distal and slightly medial to LI 1.

Prior studies [4,9,35] have investigated the use of this point in the treatment of xerostomia. From a TCM perspective, it is considered a modification of LI 1, a *Jing Well* point. According to TCM theory, all *Jing Well* points “clear heat.” In the case of this patient population, “excess heat” is produced by the radiation therapy.

Ear: Points on the ear include *Shenmen*, Point Zero, and Salivary Gland 2-prime. These points have been found to be effective in the treatment of xerostomia in prior studies [4,9,35]. *Shenmen* is used to alleviate pain, reduce inflammation, and calm the mind. The area where it is located is supplied by a mixed branch of the vagus, facial, and trigeminal nerves and the cervical plexus. Point Zero is also known as the vagus nerve point and is used to regulate various organ functions. Salivary Gland 2-prime is used to regulate salivary function and is supplied by the auriculotemporal branch of the trigeminal nerve as well as a mixed branch of the vagus, facial, and trigeminal nerves and the cervical plexus.

Treatment Group (active acupuncture)

36 gauge x 30 mm acupuncture needles at the following body points: CV24, K6 (bilateral), Lu7 (bilateral).

40 gauge x 15 mm acupuncture needles at LI 1-prime (bilateral) and the following ear points: Shenmen (bilateral), Point Zero (bilateral), and Salivary Gland 2-prime (bilateral)

One placebo needle at Gb 32 above the right knee (Note: This is intended to provide participants in the active treatment group with a stimulus that will not elicit *de qi* sensation).

5.2 Sham Treatment

Well-designed clinical acupuncture trials require a sham procedure that is indistinguishable from the real treatment, yet inactive. Some authors have concluded that needling anywhere on the ear may cause a physiologic response and may not be an inert procedure. Nevertheless, Usichenko and colleagues have used 3 points on the helix of the ear ipsilateral to the site of injury with success in 3 different studies [37-39]**.** Fixed sham points on each ear will be used as shown below. These points are located in the middle of the ear helix and are known as Helix 2, Helix 3, and Helix 4 [40].

Auricular sham points: 3 points on the helix of each ear (6 points total).

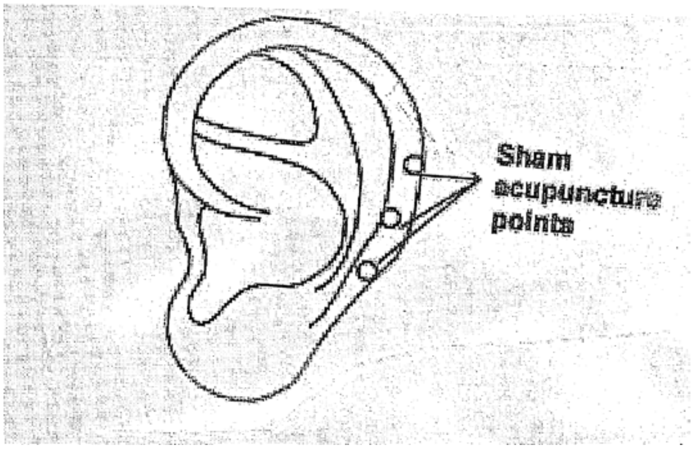

By having a placebo needle in the active group and having a real body point needle and real ear needles in the placebo group, participants will be unable to identify their group assignment. In addition, as patients will be acupuncture naïve they do not know what the "correct" feeling should be. The procedure in this trial is similar to that used by Dr. Brian Berman and colleagues for a trial that examined acupuncture for osteoarthritis of the knee [20]. However, in the Berman study although the blinding was effective in terms of the treatment procedures, the lack of benefit in terms of pain control in the placebo group increased the rate of accurate guesses in the placebo group; in essence resulting in an unblinded study. This is an inherent problem with any placebo controlled trial and especially problematic with acupuncture trials. In order to avoid this risk, patients will be told that we are examining two different forms of acupuncture compared to a usual care group. In the consent form, patients are told there are two acupuncture groups using different acupuncture points. This will help to diminish any of the demand characteristics associated with knowing they could be in a true acupuncture or sham acupuncture group. In our study, the "sham" group is getting real needles inserted at real points along with sham needles at sham points. Therefore, it is not deception to say they are getting acupuncture. Moreover, trials that have compared real and sham acupuncture versus standard care have found similar benefits of both treatments over usual care. Therefore, sham acupuncture, in this case real needles at real points, may in fact be a form of

acupuncture that results in similar outcomes to the "real" acupuncture. The design of the protocol keeps the participants blind to group assignment. Both groups are getting real and sham needles. The protocol is not compromised if the patients know they are in Group 1 versus Group 2. We will also be able to examine expectations of the benefits of the intervention at baseline and at mid- treatment. We are using this exact same technique currently in a large, multi-center, NCI R01 funded trial being conducted in Shanghai, China and at MD Anderson.

Although no "gold standard" has been established for placebo controls in acupuncture trials, ideally, non-penetrating needles placed at inactive points should be used. Park and colleagues validated a non-penetrating, telescoping needle with a separate device that attaches it to the skin [41,42]. This device is too heavy and bulky for use on auricular points, but will be used on body points in this study.

The sham treatment will be given according to the same schedule as the active acupuncture treatment. Participants in both groups will be placed in a comfortable supine position. Each point will be identified and marked on the skin. A total of 14 points will be used in both groups. The sham procedure for body points is outlined below:

Sham Group (inactive acupuncture)

Sham Location 1 - placebo needle at inactive point located 0.5 cun below and 0.5 cun lateral to CV 24 on the chin

Sham Location 2 - placebo needle at inactive point located 0.5 cun radial and 0.5 cun proximal to SJ 6 between SJ and LI Channels (bilateral UE)

Sham Location 3 - placebo needle at inactive point located 2 cun above Sham Location 2 between SJ and LI Channels and between LI7 and LI8 (bilateral UE)

Sham Location 4 - placebo needle at inactive point located 1.0 cun below and 0.5 cun lateral to St 36, between St and Gb Channels (bilateral LE)

One 32 gauge x 30mm acupuncture needle at GB32 above the right knee (Note: This point is not indicated for dry mouth and is used to elicit *de qi* sensation in the control group.)

5.3 Standard of Care

All patients will receive standard oral hygiene patient teaching information that includes instructions regarding mouth rinses, use of lip balms, use of mild fluoride toothpaste, the importance of adequate oral hydration, and other standard advice. All patients should continue with standard oral hygiene throughout the study. Each participating site determines the standard oral hygiene recommendations used at their site. All efforts should be made to ensure standard oral hygiene remains consistent across patients enrolled at the participating site.

5.4 Needling/Training Procedures

The acupuncture needles are made by Seirin, Kyoto, Japan. The manufacturer conforms to the requirements of the ISO 9002, EN46002 and CE.U.S. Food and Drug Administration's International Good Manufacturing Practices (GMP) and the World Health Organization's standards for quality and safety. The stainless steel needles used in the ear

and for one body point (LI 1-prime) are 40 gauge in diameter x 15mm in length, and the needles used for all other body points are 36 gauge x 30 mm in length. The needles with guide tubes are provided in individual sterile packages.

Acupuncture will be performed by a certified acupuncturist designated by the NCORP site. The acupuncturists who participate in this study will meet the local state acupuncture licensing requirements and have passed the National Certification Commission for Acupuncture and Oriental Medicine (NCCAOM) examination if required by state regulations. Acupuncturists in states that do not require NCCAOM certification who meet requirements to practice in their state will be allowed to participate upon submission of a copy of their state license and resume/CV. These are the accepted standards for acupuncturists and will insure their qualifications. To further ensure their acupuncture experience, they will also need to provide proof of CPR certification valid within two years, and at least one year of clinical acupuncture practice experience. All acupuncturists involved in this study will participate in a video-based training program found on the Wake Forest NCORP website and all will be required to provide evidence of certification of training in human subject protection.

The acupuncture points selected in this protocol are common and widely used for many other treatments. A properly trained and experienced acupuncturist should have no problem locating them accurately. Training courses and teaching materials will be supplied to the acupuncturists by MD Anderson Cancer Center. The training course will be concentrated on this particular protocol procedure including point selection, acupuncture needle application, length of treatment, frequency of treatment and, other protocol guidelines. For reference purposes, training materials will be made available online to participating acupuncturists.

The participating acupuncturists, who meet all of the above criteria (state acupuncture license, NCCAOM exam or meet state regulatory requirements, one year of acupuncture experience, attended MDACC training course, CPR certification, and human subject protection training), will be approved by the NCORP Research Base for acupuncture research participation. The participating acupuncturists may be an employee of the participating site or may be contracted through the participating site. Acupuncture treatments may be conducted on-site or in a private practice setting by the selected trained acupuncturist(s) that meet the above requirements.

If all requirements are met, chiropractors and licensed physicians with acupuncture credentialing may be considered for participation in the trial on an individual basis. These individuals may also be an employee of the participating site or may be contracted through the participating site.

6.0 STUDY REQUIREMENTS

6.1 Study Plan

All patients will receive standard oral hygiene instructions and complete the baseline assessments: the Xerostomia Questionnaire (XQ), the MD Anderson Symptom Inventory for Head and Neck Cancer (MDASI-HN) questionnaire, The Functional Assessment of Cancer Therapy (FACT-G), and the Acupuncture Expectancy Scale (AES) <u>*before*</u> enrollment and randomization occurs.

All patients in all groups will complete the XQ, MDASI-HN, and the FACT-G again at week 4 (±1 week). Participants in the active and sham acupuncture groups will complete the AES again at week 4. If patients have a minor response (10-19 point decrease in XQ score from

baseline), they will continue their assigned acupuncture treatment for an additional 4 weeks. Patients who have no response (increase in XQ score or decrease of <10 points from baseline), partial response (20 or more point decrease in XQ score from baseline), or complete response (XQ score = 0) will receive no further acupuncture treatment.

All patients (i.e., Standard Oral Hygiene group, and complete, minor, partial, and non-responders of the acupuncture groups) will complete the XQ, MDASI-HN, and FACT-G again at weeks 8 (±1 week), 12 (±2 weeks), and a final time at the 6 month follow-up (±1 month), and then will be taken off study. All patients will also have sialometry collection at all timepoints as well.

Patients in the Standard Oral Hygiene group or the sham acupuncture group who have completed study participation and are taken off-study will have the opportunity to receive 3 sessions of true acupuncture at no cost. These 3 sessions will be conducted in the same manner as the active true acupuncture arm using the same acupoints. These 3 free sessions will occur after the 6 month follow-up has been completed. There is no collection of patient data for these 3 free sessions.

6.2 Baseline Assessments

After the patient has signed consent and all eligibility requirements are met, the patient will complete the following before being enrolled and randomized to 1 of 3 study arms:

- Xerostomia Questionnaire (XQ)
- MD Anderson Symptom Inventory for Head and Neck Cancer (MDASI-HN) questionnaire
- The Functional Assessment of Cancer Therapy (FACT-G)
- Acupuncture Expectancy Scale (AES)
- Sialometry
- Concomitant Medications

6.3 On-Study Assessments

Please see Patient Study Calendar (Section 7.5) for complete schedule of events.

All patients will have study visits at the end of week 4 (±1 week), week 8 (±1 week), week 12 (±2 weeks) and a follow-up visit at 6 months (±1 month) after treatment (i.e. last acupuncture session received) for patients randomized to either acupuncture group or 7. 5 months (±1 month) after consent for those randomized to the standard oral hygiene group. These study visits will be conducted to assess response.

A study visit should also be completed if the patient terminates from the study early.

Standard Oral Hygiene Arm:
Patients randomized to the Standard Oral Hygiene arm will receive standard care only. Patients will complete the following at each study visit as defined above:

- Xerostomia Questionnaire (by phone if patient is unable to come to clinic for Response assessment)
- MDASI-HN Questionnaire
- FACT-G questionnaire
- Sialometry

- Concomitant Medications

True Acupuncture and Sham Acupuncture Arms:
Patients randomized to the true acupuncture and sham acupuncture arms will receive standard care in regards to their xerostomia. In addition, patients will receive either true or sham acupuncture twice weekly for four weeks.

The following will be completed at each acupuncture visit:
- Vital signs
- Review of adverse events
- Review of concomitant medications
- TAT or SAT

At the end of week 4 patients will complete:
- Xerostomia Questionnaire (by phone if patient is unable to come to clinic for Response assessment)
- MDASI-HN Questionnaire
- FACT-G questionnaire
- AES Questionnaire (week 4 only)
- Sialometry
- Adverse Events
- Concomitant Medications

At 8 and 12 weeks and at 6 months after treatment, all acupuncture patients will complete the following:

- Xerostomia Questionnaire (by phone if patient is unable to come to clinic for Response assessment)
- MDASI-HN Questionnaire
- FACT-G Questionnaire
- Sialometry
- Adverse Events
- Concomitant Medications

6.4 Off-Study Assessments
All patients taken off-study at the 6 month follow-up visit, or any other time during the study should have a final study visit and complete the XQ, MDASI-HN, FACT-G, and sialometry prior to being removed from study.

6.5 Study Compliance
Compliance is a key factor in analyzing the information collected in this trial. Patients should remain compliant with study requirements to the very best of their ability. Patients should be counseled on compliance issues and encouraged to follow the protocol as outlined. Telephone calls reminding patients of their assessment appointments should be made. Reminders should include information on the time and date of study visit, the completion of forms, and sialometry collection. Study calendars, diaries, AE assessment forms, and any other forms used to assess and collect research data should also be mentioned.

6.6 Intervention Delay
Patients who miss acupuncture sessions may have sessions made up. The sessions should be rescheduled as close as possible and no more than 4 days after the missed session. If the missed session cannot be rescheduled within 4 days, it will not be made up and the

patient will move on to the next scheduled subsequent session unless the missed session was their last protocol required session. If 2 consecutive sessions or a total of 3 or more sessions during the first 4 weeks of treatment are missed, patients will be taken off-study.

6.7 Study Follow-Up

There is no other required follow-up for this trial after the 6 month follow-up visit. However, if the treating physician feels that a follow-up is necessary on a patient, the patient may be taken off-treatment and followed until no longer felt necessary, then taken off-study.

7.0 MEASUREMENT OF EFFECT

7.1 Xerostomia Questionnaire (XQ)

The XQ is an 9-item questionnaire that has been validated in several cohorts [7,19]. Subjects rate each symptom on an 11-point ordinal Likert scale from 0 to 10, with higher scores indicating greater dryness or discomfort due to dryness. The questions are equally divided into 4 items about oral dryness while eating or chewing and 4 items about dryness while not eating or chewing. The 9th item (first question) asks about oral comfort with dentures and does not apply to the majority of patients. The XQ will be scored at the end of 4 weeks of treatment to determine response. This will be done as follows: add response values for questions 2-9 and divide by 8 to calculate mean. Then, multiply by 10 for a final summary score ranging between 0 and 100, with higher scores representing more xerostomia. See below for response definitions.

7.2 MD Anderson Symptom Inventory for Head and Neck Cancer (MDASI-HN)

Symptoms will be systematically assessed using the validated MDASI-HN [32,43]. The MDASI has been used in Phase I though Phase IV trials in the U.S. and in Europe. This tool can be easily used in clinical and research settings. The MDASI measures, on a numeric rating scale of 0-10, both the severity of symptoms and the interference symptoms cause in patients' daily activities. The 13 core MDASI symptom items are based on extensive evaluation of symptoms common to cancer and cancer treatment. Symptoms on the core MDASI include pain, fatigue, and appetite changes that the head and neck cancer population typically experiences. Patients easily complete it as a self- report tool.

The MDASI was developed so that items relevant to specific cancers and cancer treatments identified through focus groups could be added. The MDASI-HN includes 9 head and neck–specific items. The instrument was validated in a cohort of more than 200 patients and found to be highly reliable [32].

7.3 The Functional Assessment of Cancer Therapy (FACT-G)

Health-related quality of life will be assessed with the FACT-G [44]. This instrument is able to discriminate between individuals with metastatic and non-metastatic disease, as well as between patients at different stages of illness. The scale has been found to have good concurrent validity, high internal consistency (0.89), and good test-re-test reliability (0.82 to 0.88).

7.4 Acupuncture Expectancy Scale (AES)

To determine the relationship between outcome expectancy related to acupuncture and clinical response, we will use the Acupuncture Expectancy Scale. This 4-item instrument was developed by Mao et al. and found to be reliable (Cronbach's α of 0.82) and valid by positive correlation with patient self-reported efficacy and satisfaction [31]. The scale was further validated among cancer patients who were mostly acupuncture naïve[27]. Expectancy of a benefit from acupuncture appeared to be increased by educating patients on the

scientific theory and clinical evidence of acupuncture. Higher expectancy was also found in patients who had previously participated in an acupuncture trial compared with those who had not participated in an acupuncture trial[27]. In the proposed study, we will evaluate expectancy as a predictor of response to acupuncture.

Response Assessment for Xerostomia Questionnaire (XQ) Patient response will be assessed by determining XQ scores at baseline and at 4 weeks after beginning treatment. Response after 4 weeks of treatment will be calculated based on XQ score at baseline minus XQ score at 4 weeks. Duration of response will be assessed by determining XQ scores at 8 and 12 weeks and the 6 month follow-up. Patients will circle the appropriate response to the question and the corresponding numbers will be used to determine the response. The corresponding numbers will be totaled giving the patient's score.

Protocol responses are listed below:

No Response: Xerostomia worsening or decrease of < 10 points in score from baseline.

Minor Response: 10 – 19 point decrease in score from baseline score.

Partial Response: 20 point or higher decrease in score from baseline score.

Complete Response: Complete resolution of all xerostomia as reported by the patient on the XQ with a score of 0

7.5 Study Calendar of Events

Study Calendar of Events		Week 1		Week 2		Week 3		Week 4			Week 5		Week 6		Week 7		Week 8					
Assessments	Baseline (T0)	S1	S2	S1	S2	S1	S2	S1	S2	4 weeks (T1)	S1	S2	S1	S2	S1	S2	S1	S2	8 Weeks (T2)	12 Weeks (T3)	6 Months (T4)*	7.5 Months (T4)**
Informed Consent	X																					
MDASI-HN	X									X									X	X	X	X
FACT-G	X									X									X	X	X	X
Sialometry	X									X									X	X	X	X
Standard Oral Hygiene Instructions	X																					
Xerostomia Questionnaire (XQ)	X									X									X	X	X	X

Acupuncture (G1-true G2-sham):		Week 1		Week 2		Week 3		Week 4			Week 5		Week 6		Week 7		Week 8					
Vital Signs	X	X	X	X	X	X	X	X	X		X	X	X	X	X	X	X	X				
Concomitant Medications	X	X	X	X	X	X	X	X	X		X	X	X	X	X	X	X	X	X	X	X	
Adverse Events		X	X	X	X	X	X	X	X		X	X	X	X	X	X	X	X	X	X	X	
Acupuncture		X	X	X	X	X	X	X	X		X	X	X	X	X	X	X	X				
Expectancy Questionnaire (AES)	X									X												

Standard Oral Hygiene Group:		Week 1		Week 2		Week 3		Week 4			Week 5		Week 6		Week 7		Week 8					
Concomitant Medications	X									X									X	X		X
Expectancy Questionnaire (AES)	X																					

(T1) + or – 1 week (T2) + or – 1 week (T3) + or – 2 weeks (T4) * Acupuncture groups, T4 is 6 months (±1 month) after the last acupuncture treatment received.
** Standard Oral Hygiene group, T4 is 7.5 months (±1 month) after baseline.

Acupuncturist will perform the required assessments during acupuncture sessions. The acupuncturist will complete the TAT/SAT form and submit directly to WF NCORP.

Coordinators should complete a MedWatch **only** for Grades 4 and 5—definitely related, probably related or possibly related to the intervention and submit to the RB DMC.

Acupuncture treatment must begin within 30 days of Baseline data collection and enrollment.

8.0 SIALOMETRY

8.1 Saliva Flow Examination

Unstimulated Whole Saliva (USFR): Patients are instructed to refrain from eating, drinking, and dental hygiene for a minimum of 60 minutes before saliva collection. The patient is seated upright in a quiet area where he/she will remain undisturbed and is told to minimize orofacial movements. The patient will relax in the designated area for 15 minutes before saliva collection. Patients are instructed not to attempt to increase or control salivation actively (such as sucking or swallowing) but to simply relax. A collection vial (including seal and cover) will be weighed on a calibrated balance with accuracy to 0.01 gm. The patient is instructed to first clear his/her mouth by swallowing. Then, with the head held slightly forward, the patient is instructed not to talk or swallow during the 5-minute collection but to allow saliva to collect in the floor of the mouth. The 5-minute collection time is initiated on a digital timer and in view of the patient. The patient should then expectorate the accumulated saliva into the pre-weighed vial after 60 seconds. The patient should repeat this procedure 4 more times for a total collection time of 5 minutes. Patients are reminded not to swallow or talk during the entire collection period. At the end of the 5 minutes, the collection vial is promptly sealed, weighed, placed on ice and transported to the laboratory.

Stimulated Whole Saliva (SSFR): Patients are to rest for 5 minutes prior to stimulated saliva collection. The exogenous stimulant will be a neutral chewing gum from Wrigley (unflavored gum base) used previously for SSFR. The patient will chew the gum for 3 minutes, then expectorate the gum and saliva into a disposable cup. Next, the patient should swallow to clear the mouth. The patient's saliva will then be collected in the vial for 5 minutes using a method identical to USFR collection. After a 5 minute rest period, repeat (i.e. chew gum for 3 minutes, expectorate gum and saliva into a disposable cup, swallow, then collect saliva for 5 minutes as above.)

8.2 Saliva Constituent Analyses

We will determine acupuncture's effects on saliva-based factors including pH, buffering capacity, and viscosity, as well as levels of total protein, CGRP and VIP from SSFR collected at baseline, week 4, week 8, week 12 and 6 months. The value of pH and buffering capacity will be measured using a portable pH meter with micro electroprobe. Buffering capacity will be measured using the method previously described by Siqueria et al. (79). All salivary outcomes will be measured at MD Anderson from frozen samples assayed in batch to decrease variance. Viscosity of the saliva (0.5 ml) will be determined using LVT Wells-Brookfield cone-and-plate digital viscometer (Brookfield Engineering Laboratory) (80). The total amount of protein in each saliva sample will be determined using the Bradford assay (Biorad protein assay kit). Levels of CGRP and VIP will be quantified using neuropeptide-specific RIA kits (Peninsula-Bachem) (81). The concentration of peptides will be normalized by the amount of protein in each sample. All assays will be conducted in the laboratory of Dr. Peiying Yang. Once the saliva samples have been assayed they will be destroyed.

9.0 ADVERSE EVENTS AND REPORTING REQUIREMENTS

- Adverse Event/Serious Adverse Event reporting begins after the informed consent is signed.
- Serious Adverse Events occurring within 30 days of study completion must be reported via FDA Form 3500 (MedWatch).

9.1 Protocol Specific Reporting for Adverse Events (AEs)

- DEFINITION: An adverse event (AE) is any untoward medical occurrence in a study participant.

- Grades 1, 2, and 3 expected (solicited) and unexpected (unsolicited) AEs that meet the above definition for an AE and are ONLY definitely related, possibly related or probably related to the intervention should be reported to the RB DMC using the Toxicity Assessment Sheet.

- Hospitalizations for routine scheduled procedures and treatments do not need to be reported. Example: Patient admitted for gall bladder surgery.

9.2 Protocol Specific Reporting for Serious Adverse Events (SAEs)

DEFINITION: ICH Guideline E2A and Fed. Reg. 62, Oct. 7, 1997 define serious adverse events as those events which meet any of the following criteria?

- Results in death
- Is life threatening (Note: the term life-threatening refers to an event in which the patient was at risk of death at the time of the event; it does not refer to an event which hypothetically might have caused death if it were more severe).
- Requires inpatient hospitalization or prolongation of existing hospitalization
- Results in persistent or significant incapacity or substantial disruption of the ability to conduct normal life functions.
- Is a congenital abnormality/birth defect
- Events that may not meet these criteria, but which the investigator finds very unusual and/or potentially serious, will also be reported in the same manner.

- **Grades 3, 4, and 5 expected (solicited) and unexpected (unsolicited) SAEs that meet the above definition for SAEs and/or regardless of attribution (i.e. regardless of whether they are related to this study intervention or not) should be reported to the RB DMC using the FDA Form 3500 (MedWatch).**

- Site staff and/or Principal Investigators will report to the RB Data Management Staff within 24 hours of discovering the details of all unexpected severe, life-threatening (grade 4) and/or fatal adverse events (grade 5) if there is reasonable suspicion that the event was definitely, probably, or possibly related to the study intervention.

Otherwise, the MedWatch should be sent to the RB DMC by fax or email within 10 working days of discovering the details of the SAE.

Data Elements to include on the MedWatch are:

- SAE reported date
- CTCAE Term (v5.0)
- Event onset date and event ended date
- Severity grade (use table provided in Section 9.3 below)
- Attribution to study intervention (relatedness)
- Action taken with the study participant and intervention
- Outcome of the event
- Comments

9.3 Guidelines to Determine Grade and Severity of AEs and/or SAEs

The active version of the CTCAE is identified and located on the CTEP website at http://ctep.cancer.gov/protocolDevelopment/electronic_applications/ctc.htm

Identify the adverse event using the NCI Common Terminology Criteria for Adverse Events (CTCAE) version 5.0. The CTCAE provides descriptive terminology and a grading scale for each adverse event listed. A copy of the CTCAE can be found at http://ctep.cancer.gov/protocolDevelopment/electronic_applications/ctc.htm#ctc_40

AEs will be assessed according to the CTCAE grade associated with the AE term. AEs that do not have a corresponding CTCAE term will be assessed according to the general guidelines for grading used in the CTCAE v5.0. as stated below.

Grade	Severity	Description
1	Mild	Mild; asymptomatic or mild symptoms; clinical or diagnostic observations only; intervention not indicated.
2	Moderate	Moderate; minimal, local or noninvasive intervention indicated; limiting age-appropriate instrumental ADL*.
3	Severe	Severe or medically significant but not immediately life-threatening;<br>Hospitalization or prolongation of hospitalization indicated; disabling; limiting self-care ADL**.
4	Life threatening	Life-threatening consequences; urgent intervention indicated.
5	Fatal	Death related to AE.

Activities of Daily Living (ADL)

***** Instrumental ADL refer to preparing meals, shopping for groceries or clothes, using the telephone, managing money, etc.

****** Self-care ADL refer to bathing, dressing and undressing, feeding self, using the toilet, taking medications, and not bedridden.

The Research Base Grant PI, Safety and Toxicity Review Committee and/or Study Chair will take appropriate action to inform the membership and statistical personnel of any protocol modifications and/or precautionary measures, if this is warranted.

The RB DMC is responsible for communicating AEs/SAEs to the FDA, the drug sponsor, WF IRB, the WF Safety and Toxicity Review Committee (STRC) and/or other regulatory agencies as appropriate per agency reporting requirements.

Institutions must comply with their individual Institutional Review Board (IRB) policy regarding submission of documentation of adverse events. All MedWatch reports should be sent to the local IRB in accordance with the local IRB policies.

9.4 Follow-up of SAEs

Site staff should send follow-up reports as requested when additional information is available. Additional information should be entered on the MedWatch form in the appropriate format. Follow-up information should be sent to the RB Data Management Center as soon as available.

SAEs (Grade 4 and/or Grade 5) for this protocol should be followed for those related to the study intervention. Documentation should include:

- PID
- Date of SAE
- Description of the event
- Relationship of the SAE to the study intervention
- Severity
- Intervention/Resolution

If the AE is attributed to anything other than study therapy, it should be assigned the attribution of “not related”. The study team will work with the acupuncturist in gathering AEs between study visits. Acupuncturists will record AEs on the provided SAT/TAT forms and study staff will speak with the acupuncturist regarding AEs in order to determine grade and attribution. The study staff may also contact the patient directly to gather more information regarding the event.

10.0 STATISTICAL CONSIDERATIONS

The primary objective will be assessed by determining the Xerostomia Questionnaire score before and 4 weeks after starting acupuncture. The Xerostomia Questionnaire is an 9-item survey with each item scored between 0 and 10 then the final score normalized to a 0-100 scale, and has been validated in several cohorts [19,45].

We will compare the three groups using analysis of covariance (ANCOVA) adjusting for the baseline XQ score. If the test for the overall comparison is significant ($p<0.05$), we will then perform pairwise comparisons, each at a 5%/3 = 1.33% significance level, to control for the overall type I error rate. We have powered the study to be able to detect a difference of 10 points between each pair of groups on a 0 to 100 scale for the XQ. In order to detect this difference we will require 64 patients per group. This assumes a t-test with a two-sided significance level of1.33% and 84% power, and that the standard deviation of responses is about 16 [46]. Our actual ANCOVA analysis is expected to yield higher power of detecting the same differences due to the potentially reduced within-group SD of the response after controlling for the baseline XQ score. We will, in general, have higher power to detect clinically significant changes/differences in individual group XQ scores over time if these changes range, as expected, from 15-20 points [47], depending on the level of correlation between the baseline and 4-week XQ scores.

In order to allow for up to a 20% drop out rate, a total of 240 patients will be entered into this study, or 80 per group. Similar analyses will be conducted for the outcomes of MDASI-HN and FACT-G.

In patients who report a response to the acupuncture intervention at 8 weeks, an additional assessment will be made for all three instruments (XQ, MDASI-HN and FACT-G) at week 12 and 6 months after treatment (i.e. last acupuncture session received) to estimate the response. This analysis will be exploratory and its outcomes will be estimates of responses and variability of responses between patients (mean ± standard deviations) for XQ, MDASI-HN and FACT-G.

When applicable, we will also conduct linear mixed model analysis to assess the group differences over time as well as the interaction effects between group and time, controlling for

the appropriate baseline outcomes.

<u>Analysis for the secondary objectives.</u> Objective 1.
We will also summarize the duration of response up to a maximum of 6 months after treatment (i.e. last acupuncture session received) in the subgroup of patients who report a response to the acupuncture intervention and will test for differences between groups.

Objective 2-4. Analyses for these objectives will be similar to those for assessing the primary objective, except using different outcomes.

Objective 5. To assess this objective, we will preliminarily test for the interaction effects between treatment group and expectancy.

Acupuncture Expectancy Scale (AES): We will also explore the association between expectancy and outcomes using information from the Acupuncture Expectancy Scale (AES). Expectancy will be assessed among all participants using the AES at baseline. For patients randomized to either acupuncture group, it will be assessed again at week 4. The AES assessment will be correlated with severity of xerostomia using the XQ (and other continuous variables). More complex analyses will also evaluate whether expectancy moderates outcomes. Following the procedures of Baron and Kenny[48], the evaluation of moderation involves examining the interaction between intervention condition and baseline expectancy. In a series of mixed model analyses, tests of the interaction of intervention condition with expectancy will be evaluated to determine whether baseline expectancy changes the impact of acupuncture condition on the outcome measures [i.e., determine whether acupuncture (active or inactive) is more effective based on baseline expectancy].

Changes in xerostomia symptoms will also be examined as a possible mediator of the effects of acupuncture on QOL outcomes following the procedures of Baron and Kenny[48] using generalized linear mixed model regression. The mediation effect estimate will be computed according to MacKinnon[49], who describes mediation as the difference of the intervention effect on outcome with and without the presence of the mediators, or alternatively, the product of the effect of the intervention on the mediators and the effect of the mediators on the outcome controlling for the intervention condition.

11.0 STUDY ACCRUAL

240 participants are expected to be enrolled on this trial. It is expected that 5-7 participants will be enrolled each month. An accrual of 5-7 patients per month is expected since 1) certified acupuncturists are available at most of our NCORP sites, 2) interest in applying acupuncture to western medicine is escalating, 3) objective benefits of acupuncture for xerostomia have been reported, and 4) the condition lacks any significant treatment. Accrual for the trial would thus be expected to take 1.8 to 2.5 years.

12.0 DATA MANAGEMENT

Case report form submission guidelines are:

- Enrollment documents including eligibility checklist **prior to** enrollment
- Baseline forms **within 14 days** of enrollment
- Serious adverse events (SAE) **within 24 hours** of event and/or knowledge of event
- Course/cycle information **within 14 days** of completing course/cycle

- Other case report forms **within 14 days** of event.

NCORP site staff will electronically enroll their study participants in the WF NCORP RB database website, CCRBIS, at https://ccrbis.phs.wakehealth.edu

- Log in to the database website using your CCRBIS username and password.
- In the drop-down menu next to Enroll Patient/Patient Info select 97115, then click Enroll Patient/Patient Info.
- Click on Enroll New Patient.
- Complete the Eligibility Checklist/Enrollment Form then click Submit.
- Following successful submission, a confirmation page will appear with the assigned PID, **print this page for your records**.
- Submit a copy of the signed informed consent form to the WF NCORP Data Management Center (DMC) by fax at (336) 713-6476 or mail to:

WF NCORP Research Base Data Management Center
Wake Forest Baptist Medical Center
Building 525@Vine, 4th floor Medical Center Boulevard
Winston-Salem, NC 27157

If you have questions related to the subject enrollment process or require assistance with enrollment, please contact the WF NCORP Research Base DMC between 8:00am and 5:00pm EST, Monday through Friday at (336) 706-0891.

13.0 DATA MONITORING COMMITTEE

The Wake Forest NCORP Research Base has established a Data Safety Monitoring Board (DSMB) that is independent of study leadership, is free of conflicts of interest, and has formal policies and procedures approved by the NCI. The main objective of the DSMB is to:

- Ensure that patients in the clinical trial are protected
- Ensure that evaluation of interim results and decisions about continuing, modifying, or terminating a clinical trial and reporting results are made competently
- Ensure that the credibility of clinical trial reports and the ethics of clinical trial conduct are above reproach.

This DSMB will review the data from this protocol every 6 months or as requested.

14.0 SITE REGISTRATION

IRB Approval:
For CTEP and Division of Cancer Prevention (DCP) studies open to the National Clinical Trials Network (NCTN) and NCI Community Oncology Research Program (NCORP) Research Bases after March 1, 2019, all U.S.-based sites must be members of the NCI Central Institutional Review Board (NCI CIRB). In addition, U.S.-based sites must accept the NCI CIRB review to activate new studies at the site after March 1, 2019. Local IRB review will continue to be accepted for studies that are not reviewed by the CIRB, or if the study was previously open at the site under the local IRB. International sites should continue to submit Research Ethics Board (REB) approval to the CTSU Regulatory Office following country-

specific regulations.
Sites using their local IRB or REB, must submit their approval via email to the Lead Protocol Office, the Wake Forest NCORP Research Base to NCORP@wakehealth.edu.

Additional Requirements:

Additional requirements to obtain an approved site registration status include:

- An active Federal Wide Assurance (FWA) number;
- An active roster affiliation with the Lead Protocol Organization (LPO) or a Participating Organization (PO); and
- Compliance with all protocol-specific requirements (PSRs)

15.0 REFERENCES

1. Dreizen S, Brown LR, Handler S, et al. Radiation-induced xerostomia in cancer patients. Effect on salivary and serum electrolytes. Cancer 1976; **38**(1): 273-8.
2. Dreizen S, Daly TE, Drane JB, et al. Oral complications of cancer radiotherapy. Postgrad Med 1977; **61**(2): 85-92.
3. Bertram U. Xerostomia. Clinical aspects, pathology and pathogenesis. Acta Odontol Scand 1967; **25**: Suppl 49:1-126.
4. Johnstone PA, Niemtzow RC, and Riffenburgh RH. Acupuncture for xerostomia: clinical update. Cancer 2002; **94**(4): 1151-6.
5. Lin SC, Jen YM, Chang YC, et al. Assessment of xerostomia and its impact on quality of life in head and neck cancer patients undergoing radiotherapy, and validation of the Taiwanese version of the xerostomia questionnaire. J Pain Symptom Manage 2008; **36**(2): 141-8.
6. Jemal A, Siegel R, Ward E, et al. Cancer statistics, 2009. CA Cancer J Clin 2009; **59**(4): 225-49.
7. Eisbruch A, Kim HM, Terrell JE, et al. Xerostomia and its predictors following parotid-sparing irradiation of head-and-neck cancer. Int J Radiat Oncol Biol Phys 2001; **50**(3): 695-704.
8. Eisbruch A, Rhodus N, Rosenthal D, et al. The prevention and treatment of radiotherapy - induced xerostomia. Semin Radiat Oncol 2003; **13**(3): 302-8.
9. Johnstone PA, Peng YP, May BC, et al. Acupuncture for pilocarpine-resistant xerostomia following radiotherapy for head and neck malignancies. Int J Radiat Oncol Biol Phys 2001; **50**(2): 353-7.
10. Blom M, Dawidson I, Fernberg JO, et al. Acupuncture treatment of patients with radiation-induced xerostomia. Eur J Cancer B Oral Oncol 1996; **32B**(3): 182-90.
11. Blom M and Lundeberg T. Long-term follow-up of patients treated with acupuncture for xerostomia and the influence of additional treatment. Oral Dis 2000; **6**(1): 15-24.
12. Rydholm M and Strang P. Acupuncture for patients in hospital-based home care suffering from xerostomia. J Palliat Care 1999; **15**(4): 20-3.
13. Dawidson I, Angmar-Mansson B, Blom M, et al. Sensory stimulation (acupuncture) increases the release of calcitonin gene-related peptide in the saliva of xerostomia sufferers. Neuropeptides 1999; **33**(3): 244-50.
14. Dawidson I, Angmar-Mansson B, Blom M, et al. Sensory stimulation (acupuncture) increases the release of vasoactive intestinal polypeptide in the saliva of xerostomia sufferers. Neuropeptides 1998; **32**(6): 543-8.
15. Andersen SW and Machin D. Acupuncture treatment of patients with radiation-induced xerostomia. Oral Oncol 1997; **33**(2): 146-7.
16. Blom M, Lundeberg T, Dawidson I, et al. Effects on local blood flux of acupuncture stimulation used to treat xerostomia in patients suffering from Sjogren's syndrome. J Oral Rehabil 1993; **20**(5): 541-8.
17. Dawidson I, Angmar-Mansson B, Blom M, et al. The influence of sensory stimulation (acupuncture) on the release of neuropeptides in the saliva of healthy subjects. Life Sci 1998; **63**(8): 659-74.
18. Garcia MK, Chiang JS, Cohen L, et al. Acupuncture for radiation-induced xerostomia in patients with cancer: a pilot study. Head Neck 2009; **31**(10): 1360-8.
19. Pacholke HD, Amdur RJ, Morris CG, et al. Late xerostomia after intensity-modulated radiation therapy versus conventional radiotherapy. Am J Clin Oncol 2005; **28**(4): 351-8.
20. Berman BM, Lao L, Langenberg P, et al. Effectiveness of acupuncture as adjunctive therapy in osteoarthritis of the knee: a randomized, controlled trial. Ann Intern Med 2004; **141**(12): 901-10.
21. Furlan AD, van Tulder MW, Cherkin DC, et al. Acupuncture and dry-needling for low back pain. Cochrane Database Syst Rev 2005(1): CD001351.
22. Ezzo JM, Richardson MA, Vickers A, et al. Acupuncture-point stimulation for chemotherapy-induced nausea or vomiting. Cochrane Database Syst Rev 2006(2): CD002285.
23. Lee A and Done ML. Stimulation of the wrist acupuncture point P6 for preventing postoperative nausea and vomiting. Cochrane Database Syst Rev 2004(3): CD003281.
24. Proctor ML, Smith CA, Farquhar CM, et al. Transcutaneous electrical nerve stimulation and acupuncture for primary dysmenorrhoea. Cochrane Database Syst Rev 2002(1): CD002123.
25. Ezzo J, Berman B, Hadhazy VA, et al. Is acupuncture effective for the treatment of chronic pain? A systematic review. Pain 2000; **86**(3): 217-25.
26. Manheimer E, White A, Berman B, et al. Meta-analysis: acupuncture for low back pain. Ann Intern Med 2005; **142**(8): 651-63.
27. Mao JJ. Validation of acupuncture expectancy scale in cancer patients". North American Research Conference on Complementary and Integrative Medicine [Abstract], 5/09, Minneapolis, MN. 2009.

28. Crow R, Gage H, Hampson S, et al. The role of expectancies in the placebo effect and their use in the delivery of health care: a systematic review. Health Technol Assess 1999; **3**(3): 1-96.
29. Birch S and Jamison RN. Controlled trial of Japanese acupuncture for chronic myofascial neck pain: assessment of specific and nonspecific effects of treatment. Clin J Pain 1998; **14**(3): 248-55.
30. Kalauokalani D, Cherkin DC, Sherman KJ, et al. Lessons from a trial of acupuncture and massage for low back pain: patient expectations and treatment effects. Spine (Phila Pa 1976) 2001; **26**(13): 1418-24.
31. Mao JJ, Armstrong K, Farrar JT, et al. Acupuncture expectancy scale: development and preliminary validation in China. Explore (NY) 2007; **3**(4): 372-7.
32. Chambers MS, Rosenthal DI, and Weber RS. Radiation-induced xerostomia. Head Neck 2007; **29**(1): 58-63.
33. Pocock SJ. Clinical Trials: A Practical Approach. New York: John Wiley & Sons, 1983.
34. Deadman P, Al-Khafaji M, and Baker K. A Manual of Acupuncture. East Sussex, England: Journal of Chinese Medicine Publications, 1999.
35. Johnstone PA, Polston GR, Niemtzow RC, et al. Integration of acupuncture into the oncology clinic. Palliat Med 2002; **16**(3): 235-9.
36. Deng L, Gan Y, He S, et al. Chinese Acupuncture and Moxibustion, ed. Y. Cheng. Beijing: Foreign Languages Press, 1997.
37. Usichenko TI, Dinse M, Hermsen M, et al. Auricular acupuncture for pain relief after total hip arthroplasty - a randomized controlled study. Pain 2005; **114**(3): 320-7.
38. Usichenko TI, Dinse M, Lysenyuk VP, et al. Auricular acupuncture reduces intraoperative fentanyl requirement during hip arthroplasty--a randomized double-blinded study. Acupunct Electrother Res 2006; **31**(3-4): 213-21.
39. Usichenko TI, Hermsen M, Witstruck T, et al. Auricular Acupuncture for Pain Relief after Ambulatory Knee Arthroscopy-A Pilot Study. Evid Based Complement Alternat Med 2005; **2**(2): 185-89.
40. Huang LC. Auriculotherapy: Diagnosis and Treatment. Bellaire, Texas: Longevity Press, 1996.
41. Park J. Sham needle control needs careful approach. Pain 2004; **109**(1-2): 195-6; author reply 97-9.
42. Park J, White A, Stevinson C, et al. Validating a new non-penetrating sham acupuncture device: two randomised controlled trials. Acupunct Med 2002; **20**(4): 168-74.
43. Cleeland CS, Mendoza TR, Wang XS, et al. Assessing symptom distress in cancer patients: the M.D. Anderson Symptom Inventory. Cancer 2000; **89**(7): 1634-46.
44. Cella DF, Tulsky D, Gray G, et al. The Functional Assessment of Cancer Therapy Scale: Development and validation of the general measure. Journal of Clinical Oncology 1993; **11**: 570-79.
45. Eisbruch A, Rhodus N, Rosenthal D, et al. How should we measure and report radiotherapy-induced xerostomia? Semin Radiat Oncol 2003; **13**(3): 226-34.
46. Sloan JA, Frost MH, Berzon R, et al. The clinical significance of quality of life assessments in oncology: a summary for clinicians. Support Care Cancer 2006; **14**: 998.
47. EAST 5, version 5.0, Cytel Inc. 2007.
48. Baron RM and Kenny DA. The moderator-mediator variable distinction in social psychology research: Conceptual, strategic, and statistical considerations. J Pers soc Psychol 1986; **51**: 1173-82.
49. MacKinnon DP. Analysis of mediating variables in prevention and intervention research. NIDA Res Monogr 1994; **139**: 127-53.
