# Supplementary material for: Acupuncture for Chronic Radiation-Induced Xerostomia in Head and Neck Cancer: A Multicenter Randomized Clinical Trial
Source: JAMA Netw Open. 2024 May 13;7(5):e2410421. doi: 10.1001/jamanetworkopen.2024.10421 (PMC11091764; doi:10.1001/jamanetworkopen.2024.10421)
Supplement: Supplement 2. — eTable 1. Raw Means and Standard Deviations for All Outcomes Listed by Treatment Group and Time eTable 2. Multiple Imputation Sensitivity Analysis for Least Square Mean Estimates With 95% Confidence Intervals for Each Treatment Group by Time [file jamanetwopen-e2410421-s002.pdf]

Supplementary Online Content

Cohen L, Danhauer SC, Garcia MK, et al. Effectiveness of acupuncture for chronic radiation-induced xerostomia in head and neck cancer: a randomized clinical trial. *JAMA Netw Open*. 2024;7(5):e2410421. doi:10.1001/jamanetworkopen.2024.10421

eTable 1. Raw Means and Standard Deviations for All Outcomes Listed by Treatment Group and Time

eTable 2. Multiple Imputation Sensitivity Analysis for Least Square Mean Estimates With 95% Confidence Intervals for Each Treatment Group by Time

This supplementary material has been provided by the authors to give readers additional information about their work.

© 2024 Cohen L et al. *JAMA Network Open.*

eTable 1. Raw means and standard deviations for all outcomes listed by treatment group and time.

		Combined	TA	SA	SOH
**Raw Scores**	**Time Week**	**Mean (SD)**	**Mean (SD)**	**Mean (SD)**	**Mean (SD)**
**XQ score**	0	62.7 (17.1)	63.0 (17.1)	62.1 (17.5)	63.2 (16.8)
	4	54.0 (18.6)	51.1 (19.2)	54.5 (18.5)	57.3 (17.5)
	8	51.1 (19.9)	48.3 (20.4)	50.8 (20.2)	54.8 (18.6)
	12	50.7 (21.2)	48.6 (23.0)	49.1 (20.8)	54.6 (19.2)
	26	49.7 (20.5)	48.7 (21.5)	47.5 (21.1)	53.3 (18.4)
**Fact-G Total**	0	97.1 (15.5)	98.8 (15.5)	97.7 (15.1)	94.8 (15.9)
	4	99.8 (14.4)	101.9 (14.3)	100.1 (13.5)	96.4 (15.1)
	8	100.5 (13.2)	102.6 (13.1)	100.0 (12.2)	98.6 (14.4)
	12	99.1 (14.2)	103.4 (11.8)	97.8 (13.2)	95.5 (16.6)
	26	100.3 (14.6)	102.0 (14.2)	99.7 (14.5)	98.8 (15.2)
**AES Score**	0	12.1 (4.0)	11.9 (4.1)	11.7 (3.7)	12.7 (4.3)
	4	10.7 (4.2)	10.8 (4.3)	10.6 (4.1)	n/a**

TA = True Acupuncture; SA = Sham Acupuncture; SOH = Standard Oral Hygiene; XQ = Xerostomia Questionnaire; AES = Acupuncture Expectancy Scale
**AES Scores were not collected for SOH treatment group at week 4 per protocol

© 2024 Cohen L et al. *JAMA Network Open.*

eTable 2. Multiple imputation sensitivity analysis for least square mean estimates with 95% confidence intervals for each treatment group by time

	..	TA	SA	SOH	Pairwise Comparisons		
..	**Time Week**	**LS Mean 95% CI**	**LS Mean 95% CI**	**LS Mean 95% CI**	**TA v SOH p-value**	**SA v SOH p-value**	**TA v SA p-value**
**XQ Score**	4	51.1 (47.9,54.3)	54.9 (51.5,58.3)	56.5 (52.8,60.3)	0.03	0.51	0.12
	8	49.1 (45.3,52.9)	51.6 (47.8,55.5)	55.5 (52.8,59.7)	0.05	0.28	0.36
	12	49.2 (45.3,53.1)	50.4 (46.4,54.4)	54.2 (50.0,58.4)	0.09	0.19	0.68
	26	49.6 (45.6,53.7)	49.1 (45.0,53.2)	53.6 (49.4,57.9)	0.18	0.13	0.85
**FACT-G Total**	4	100.7 (98.7,102.7)	99.7 (97.6,101.8)	97.5 (95.1,99.9)	0.04	0.17	0.49
	8	100.8 (98.4,103.2)	99.5 (97.2,101.9)	98.9 (96.3,101.5)	0.29	0.74	0.46
	12	101.5 (99.1,104.0)	97.5 (95.0,99.9)	96.8 (94.2,99.4)	0.009	0.73	0.02
	26	100.0 (97.4,102.7)	99.0 (96.3,101.7)	98.9 (96.0,101.7)	0.56	0.95	0.60
**AES Score**	4	10.7 (10.0,11.5)	10.9 (10.1,11.7)	n/a**	n/a**	n/a**	0.75

Models used monotone regression with 1000 iterations and utilized baseline outcome, age, sex, race, ethnicity, and job status to impute outcomes, with an unstructured covariance.
TA = True Acupuncture; SA = Sham Acupuncture; SOH = Standard Oral Hygiene; XQ = Xerostomia Questionnaire; AES = Acupuncture Expectancy Scale;
**AES Scores were not collected for SOH treatment group at week 4 per protocol

© 2024 Cohen L et al. *JAMA Network Open.*
