# Supplementary material for: Acupuncture for Chronic Radiation-Induced Xerostomia in Head and Neck Cancer: A Multicenter Randomized Clinical Trial
Source: JAMA Netw Open. 2024 May 13;7(5):e2410421. doi: 10.1001/jamanetworkopen.2024.10421 (PMC11091764; doi:10.1001/jamanetworkopen.2024.10421)
Supplement: Supplement 3. — Data Sharing Statement [file jamanetwopen-e2410421-s003.pdf]

Data Sharing Statement

Cohen. Acupuncture for Chronic Radiation-Induced Xerostomia in Head and Neck Cancer. *JAMA Netw Open*. Published May 13, 2024. doi:10.1001/jamanetworkopen.2024.10421

Data
Data available: Yes
Data types: Deidentified participant data
How to access data: Wake Forest NCORP
When available: With publication

Supporting Documents
Document types: None

Additional Information
Who can access the data: researchers whose proposed use of the data has been approved
Types of analyses: for any purpose or for a specified purpose outside the scope of the trial
Mechanisms of data availability: with a signed data access agreement
